# Association between maternal age at childbirth and child and adult outcomes in the offspring: a prospective study in five low-income and middle-income countries (COHORTS collaboration)

**DOI:** 10.1016/S2214-109X(15)00038-8

**Published:** 2015-07

**Authors:** Caroline H D Fall, Harshpal Singh Sachdev, Clive Osmond, Maria Clara Restrepo-Mendez, Cesar Victora, Reynaldo Martorell, Aryeh D Stein, Shikha Sinha, Nikhil Tandon, Linda Adair, Isabelita Bas, Shane Norris, Linda M Richter, COHORTS investigators

**Affiliations:** a MRC Lifecourse Epidemiology Unit, University of Southampton, Southampton, UK; b Sitaram Bhartia Institute of Science and Research, New Delhi, India; c Universidade Federal de Pelotas, Capão do Leão, Pelotas, Brazil; d Hubert Department of Global Health, Rollins School of Public Health, Emory University, Atlanta, GA, USA; e All India Institute of Medical Sciences, New Delhi, India; f Department of Nutrition, University of North Carolina at Chapel Hill, Chapel Hill, NC, USA; g Office of Population Studies Foundation, University of San Carlos, Cebu, Philippines; h Medical Research Council Developmental Pathways for Health Research Unit, Witwatersrand University, Johannesburg, South Africa; i Human Sciences Research Council, Durban, South Africa

## Abstract

**Background:**

Both young and advanced maternal age is associated with adverse birth and child outcomes. Few studies have examined these associations in low-income and middle-income countries (LMICs) and none have studied adult outcomes in the offspring. We aimed to examine both child and adult outcomes in five LMICs.

**Methods:**

In this prospective study, we pooled data from COHORTS (Consortium for Health Orientated Research in Transitioning Societies)—a collaboration of five birth cohorts from LMICs (Brazil, Guatemala, India, the Philippines, and South Africa), in which mothers were recruited before or during pregnancy, and the children followed up to adulthood. We examined associations between maternal age and offspring birthweight, gestational age at birth, height-for-age and weight-for-height *Z* scores in childhood, attained schooling, and adult height, body composition (body-mass index, waist circumference, fat, and lean mass), and cardiometabolic risk factors (blood pressure and fasting plasma glucose concentration), along with binary variables derived from these. Analyses were unadjusted and adjusted for maternal socioeconomic status, height and parity, and breastfeeding duration.

**Findings:**

We obtained data for 22 188 mothers from the five cohorts, enrolment into which took place at various times between 1969 and 1989. Data for maternal age and at least one outcome were available for 19 403 offspring (87%). In unadjusted analyses, younger (≤19 years) and older (≥35 years) maternal age were associated with lower birthweight, gestational age, child nutritional status, and schooling. After adjustment, associations with younger maternal age remained for low birthweight (odds ratio [OR] 1·18 (95% CI 1·02–1·36)], preterm birth (1·26 [1·03–1·53]), 2-year stunting (1·46 [1·25–1·70]), and failure to complete secondary schooling (1·38 [1·18–1·62]) compared with mothers aged 20–24 years. After adjustment, older maternal age remained associated with increased risk of preterm birth (OR 1·33 [95% CI 1·05–1·67]), but children of older mothers had less 2-year stunting (0·64 [0·54–0·77]) and failure to complete secondary schooling (0·59 [0·48–0·71]) than did those with mothers aged 20–24 years. Offspring of both younger and older mothers had higher adult fasting glucose concentrations (roughly 0·05 mmol/L).

**Interpretation:**

Children of young mothers in LMICs are disadvantaged at birth and in childhood nutrition and schooling. Efforts to prevent early childbearing should be strengthened. After adjustment for confounders, children of older mothers have advantages in nutritional status and schooling. Extremes of maternal age could be associated with disturbed offspring glucose metabolism.

**Funding:**

Wellcome Trust and the Bill & Melinda Gates Foundation.

## Introduction

Young maternal age at childbearing (≤19 years) is associated with an increased risk of preterm birth and intrauterine growth restriction, infant mortality, and child undernutrition.^1–12^ These associations result from behavioural, social, and biological factors. Younger mothers might breastfeed for a shorter duration than older mothers^3,13^ and be behaviourally immature and therefore less able to attend to their infant's needs. They tend to have lower socioeconomic status, less schooling, and less stable partnerships than older mothers. If still growing, their nutritional needs compete with those of the fetus.^14^

Advanced maternal age (≥35 years) is associated with increased stillbirths, preterm births, intrauterine growth restriction, as well as young maternal age, and chromosomal abnormalities.^15–18^ Again, these consequences result from multiple factors. In some settings, older mothers have lower socioeconomic status, less schooling, and higher parity, whereas in others they are educated women who have delayed pregnancy for career reasons. Older mothers are at increased risk of obesity, diabetes, hypertension, and associated pregnancy complications.

Recent interest has grown in the developmental origins of human capital and adult disease, especially cardiovascular disease and its risk factors hypertension and diabetes.^19^ Studies from high-income countries show lower educational achievement in children of younger mothers.^20^ Some studies have further shown increased childhood blood pressure in the offspring of older mothers.^21–24^ No published studies have examined associations between maternal age and adult risk factors for cardiovascular disease.

Most studies linking maternal age to child outcomes are from high-income countries (HICs). However, young maternal age is more common in low-income and middle-income countries (LMICs; fertility rate among women aged younger than 19 years roughly 103 per 1000 women per year) than in HICs (21 per 1000).^25^ More young mothers in LMICs could be physically immature because of delayed completion of growth among undernourished girls. Confounding effects of socioeconomic status might differ in LMICs—for example, in some settings, young age at marriage is a norm and not necessarily associated with deprivation.^3,10^

We aimed to explore associations between maternal age and birth outcomes, child nutritional status and schooling, and adult size, body composition, and cardiometabolic risk factors. We adjusted for socioeconomic confounding factors, and explored maternal height, breastfeeding duration, and parity as potential mediators of associations.

## Methods

### Study design

COHORTS (Consortium for Health Orientated Research in Transitioning Societies) is a collaboration of five birth cohorts from LMICs, in which mothers were recruited before or during pregnancy, and the children followed up to adulthood.^26^ In this prospective study, the cohorts include the 1982 Pelotas Cohort (Brazil); the Institute of Nutrition of Central America and Panama Nutrition Trial Cohort (Guatemala); the New Delhi Cohort (India); the Cebu Longitudinal Health and Nutrition Survey (Philippines); and the Birth to Twenty Cohort (South Africa; appendix p 1).^26^ All studies were approved by appropriate institutional ethics committees. Informed verbal or written consent was obtained at recruitment from the mothers in the original birth cohort studies. Informed verbal or written consent was obtained at each round of follow-up, from a parent for childhood follow-ups and from the cohort member themselves for the adult follow-up.

### Procedures

The mother's age at the birth of the index child was calculated from data obtained at interview before or during pregnancy, or, for South African data, from birth notification forms.

Birthweight was measured by researchers (Brazil, India, and Guatemala) or obtained from hospital records (the Philippines and South Africa). Gestational age was calculated from the last menstrual period date obtained by prospective surveillance (Guatemala and India), from the mother at recruitment (the Philippines), or from medical records (Brazil and South Africa). In the Philippines, gestational age was obtained for low birthweight babies by newborn clinical assessment.^27^ Low birthweight was defined as <2500 g, preterm birth as gestational age <37 weeks, and smallness-for-gestational-age as birthweight below the age-specific and sex-specific 10th percentile of a US reference population.^28^

In all sites, post-natal weight and height were measured longitudinally with standardised methods. Measurements at 2 years were available in all sites, and 4-year measurements in all except the Philippines, where the next available age (8·5 years) was used to define so-called mid-childhood size. Height-for-age and weight-for-height were converted into *Z*-scores (HAZ and WHZ, respectively) with the WHO growth reference. Stunting and wasting were defined as HAZ and WHZ below −2 SDs, respectively. Breastfeeding data were recorded prospectively with different methods in each cohort; for this analysis we used duration in months of any breastfeeding (as opposed to exclusive breastfeeding), which was available for all cohorts except India.

Height, weight, and waist circumference were measured with standardised techniques. Fat and fat-free mass were measured with site-specific methods, as previously described.^29^ In Brazil, bioelectrical impedance was measured and results corrected based on a validation study that used isotopic methods; data are only available for men. In Guatemala, weight, height, and abdominal circumference were measured and entered into a hydrostatic-weighing validated equation. The Indian and Filipino cohorts used published equations that have been validated for use in Asian populations for estimation of body fat from skinfold measures. South Africa used dual X-ray absorptiometry (Hologic Delphi, Bedford, MA, USA). Fat mass was calculated as: % body fat × body weight, and fat-free mass as weight–fat mass.

Overweight was defined as a body-mass index (BMI) of 25 kg/m^2^ or more and obesity as BMI 30 kg/m^2^ or more. Blood pressure was measured seated, after a 5–10 min rest, with appropriate cuff sizes and a variety of devices, as previously described.^30^ High blood pressure was defined as systolic blood pressure 130 mm Hg or greater or diastolic blood pressure 85 mm Hg or greater.^31^ Fasting glucose was measured in all sites except Brazil, where a random sample was collected and glucose values adjusted for time since the last meal.^32^ Impaired fasting glucose was defined as a fasting glucose concentration of 6·1 mmol/L or greater but less than 7·0 mmol/L and diabetes as a concentration of 7·0 mmol/L or greater.^33^ Pregnant women were excluded from all these analyses.

Socioeconomic status was considered a potential confounding factor and was assessed with five variables: maternal schooling, marital status, wealth index, urban or rural residence, and ethnic origin. Wealth index was a score derived in each cohort based on type of housing and ownership of household assets (appendix p 2). The Brazilian, Indian, and South African cohorts are urban, and the Guatemalan cohort is rural; in the mixed Filipino cohort, a so-called urbanicity index was used.^34^ Brazil and South Africa had white, black, Asian, and other ethnic subgroups. Maternal height was potentially both a confounder of maternal age effects (due to secular trends in height) and a mediator (due to younger mothers not having attained final height). Maternal parity and breastfeeding duration were potential mediators (older mothers tend to have higher parity and younger mothers might breastfeed for a shorter time). Parity was coded as 1, 2, 3, or 4 or more.

### Statistical analysis

The number of offspring for whom outcomes were available diminished with age at follow-up; for example, n=17  903 (81%) for birthweight and n=10 376 (47%) for adult blood pressure. The maternal age distribution was similar in successive waves of follow-up (appendix p 3). For each outcome, we used the maximum sample with available data. To test the representativeness of our analysis sample, we compared maternal age in those included in the analysis (with data for any childhood or adult outcome of interest) and those not included, using *t* tests (appendix p 4). We used maternal age as a continuous variable where possible, but used categories (≤19 years, ≥35 years, and 5-year intervening bands) to check for linearity and for tables, figures, and odds ratio calculations. Percentage of body fat and wealth index were non-normally distributed and were Fisher-Yates transformed.^35^

We first analysed associations between maternal age and outcomes in each cohort with multiple linear regression for continuous outcomes and multiple logistic regression for dichotomous outcomes. We assessed non-linear associations with quadratic terms. We then produced pooled analyses, including main effects for each cohort and interaction terms for cohort and control variables. We tested for heterogeneity among cohorts with *F* tests, comparing sums of squares explained when effects were or were not allowed to vary across sites.^35^ We used a sequence of regression models: (1) adjusted for sex, and adult age (adult outcomes only); (2) further adjusted for socioeconomic variables; (3) further adjusted for maternal height; (4) further adjusted for breastfeeding duration; and (5) further adjusted for parity. Missing maternal wealth and schooling values were imputed with regression analysis of known values on other socioeconomic variables. Missing maternal height values were not imputed, and a dummy variable (0=not missing; 1=missing) to represent missing value was included in regression models. The Guatemala cohort is based on a randomised controlled trial of a protein and energy supplement for pregnant women and children;^26^ it comprises children living in the trial villages who were born or were younger than 7 years of age at any time between 1969 and 1977. We tested for interactions between maternal age and intervention group in this cohort, but noted no consistent evidence of interactions. In Guatemala, the 2344 participants came from 768 families; in India, the 5395 came from 5313 families; there were no siblings in the other cohorts. We used linear mixed modelling to assess whether siblings affected the associations of outcomes with maternal age, and found that they made little difference (appendix p 9). We therefore present our findings without adjustment for sibships. All analyses were done with SPSS version 21 and Stata version 12.

### Role of the funding source

The funders had no role in the study design or conduct; the management, analysis, or interpretation of the data; the preparation, review, or approval of the report; or the decision to submit the manuscript for publication. CO and CHDF had full access to all the data and take responsibility for the integrity of the data and the accuracy of the data analysis. CHDF had the responsibility to submit the report for publication.

## Results

We obtained data for 22 188 mothers from the five cohorts, enrolment into which took place at various times between 1969 and 1989. Data for maternal age and at least one outcome were available for 19 403 offspring (87%). Table 1 shows the characteristics of the participants. Mean (range) maternal age was 26 years (12–49), which was similar in all cohorts; 20–24 years was the most numerous category (appendix p 2).

44% of mothers in South Africa were married and more than 90% were married in the other cohorts (table 1). Mean maternal schooling ranged from 1·3 years (Guatemala) to 9·5 years (South Africa). 55% of mothers in Guatemala were of parity 4 or higher compared with about 35% in India and the Philippines and 16% in Brazil and South Africa. Associations between the confounding or mediating variables and maternal age and study outcomes varied among the cohorts (table 2). Older maternal age was associated with being married, having less schooling but greater wealth, longer breastfeeding duration, and higher parity. Maternal height was positively related to maternal age in the Philippines, and we noted an inverted U-shaped relation in the other cohorts, with shorter height in mothers younger than 19 years and 35 years or older (non-linear data not shown). Maternal married status, higher schooling and wealth, white ethnic origin (Brazil and South Africa), urbanicity (the Philippines), and taller height were associated with higher birthweight, child size, and schooling. Blood pressure was highest in black participants and lowest in Asians (Brazil and South Africa).

**Figure 1:**
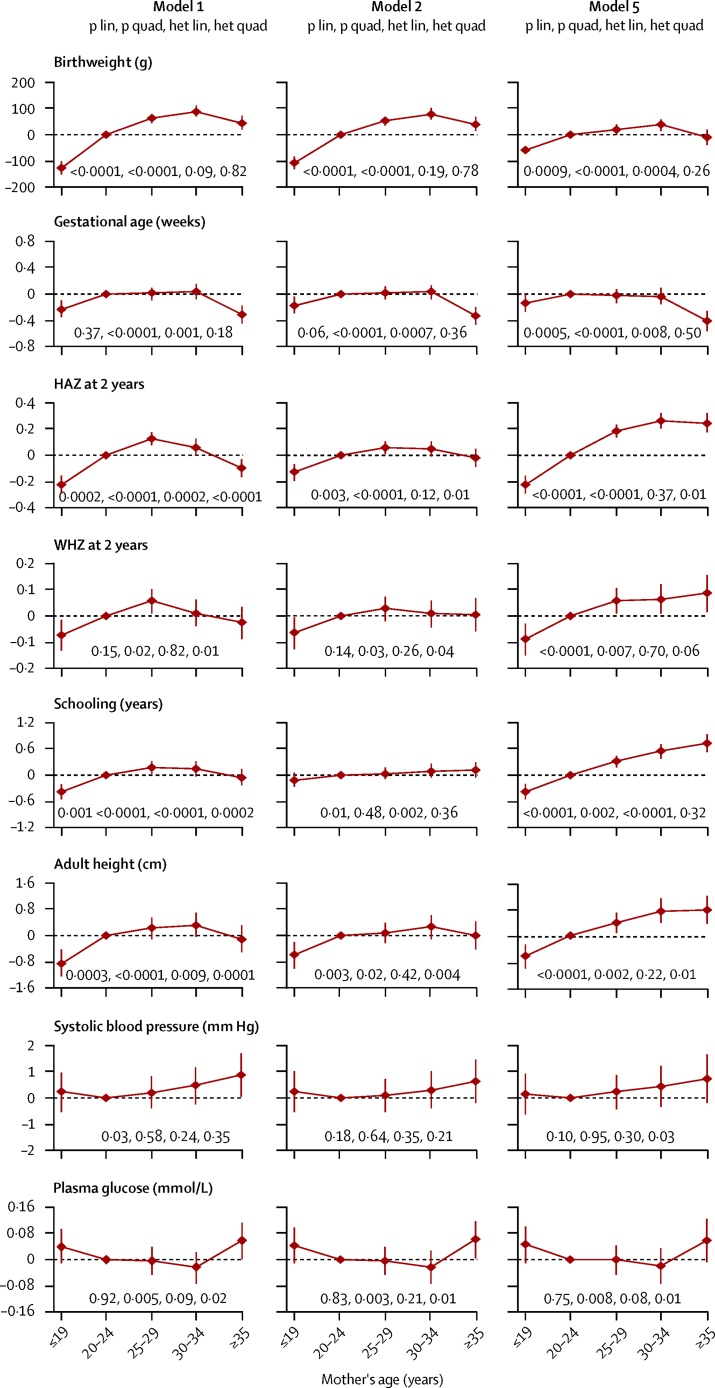
Associations between maternal age and birthweight, gestational age at birth, 2-year height and weight-for-height *Z* scores, years of schooling, adult height, systolic blood pressure, and fasting plasma glucose Each point represents the amount by which the outcome differs from the value obtained for offspring of mothers aged 20–24 years. These estimates are shown with 95% CIs, and they are obtained by pooling across all five studies. Three of the five models discussed in the text are included. Model 1 is adjusted for sex and age. Model 2 is further adjusted for maternal marital status, schooling, wealth, ethnic origin, and urbanicity. Models 3 and 4, in which further adjustment is made for maternal height and breastfeeding duration, respectively, are not shown because the results were similar to model 2. Model 5 is further adjusted for parity. Four p values are shown: p lin is from a test for linear trend in the outcome with mother's age; p quad is from a test for quadratic trend in the outcome with mother's age; het lin is the *F* test p value for heterogeneity in the linear trends in the five studies; and het quad is the *F* test p value for heterogeneity in the quadratic trends in the five studies. All four p values are derived with maternal age as a continuous variable. HAZ=height-for-age *Z* score. WHZ=weight-for-height *Z* score.

Birthweight rose with increasing maternal age, with a downturn among the oldest mothers (figure 1). Adjustment for socioeconomic factors, maternal height, and breastfeeding made little difference to this pattern. Adjustment for maternal parity attenuated the association, although maternal age 19 years or younger remained associated with lower birthweight (table 3). This association was similar for smallness-for-gestational-age (OR 1·10, 95% CI 0·94–1·24, in the fully adjusted model). Maternal age showed an inverted U-shaped association with gestational age at birth, which changed very little with adjustment (figure 1). Gestational age at birth was shorter by 0·1 weeks (95% CI 0·0–0·3) in mothers aged 19 years and younger and 0·4 weeks (95% CI 0·3–0·6) in 35 years and older in the fully adjusted model. Odds ratios for preterm birth were 1·26 (95% CI 1·03–1·53) for mothers aged 19 years and younger and 1·33 (1·05–1·67) for mothers 35 years and older (table 3). These associations showed no site–sex heterogeneity (figure 1; appendix pp 5, 6, 10, 11).

Maternal age also showed inverted U-shaped associations with 2-year HAZ and WHZ, mid-childhood HAZ, attained schooling, and adult height (figure 1; appendix p 5). Adjustment for socioeconomic factors attenuated these associations, although maternal age 19 years or younger remained associated with lower values. Adjustment for maternal height and breastfeeding made little difference. Adjustment for parity changed the shape of the associations (figure 1); although young maternal age remained associated with poorer outcomes, maternal age 25 years or older was associated with better outcomes. In the fully adjusted model, 2-year HAZ was lower by 0·23 (95% CI 0·16–0·29) for maternal age 19 years or younger, and higher by 0·24 (95% CI 0·17–0·32) for maternal age 35 years or older. Corresponding data for schooling were −0·38 years (95% CI −0·54 to −0·21) and +0·72 years (95% CI 0·53 to 0·92) and for adult height −0·63 cm (−1·00 to −0·26) and +0·77 cm (0·34 to 1·20). The associations did not change much after further adjustment for birthweight. We noted similar findings for 2-year stunting and wasting, and failure to complete secondary schooling (table 3). Substantial site–sex heterogeneity was identified for these outcomes, mainly because of more linear associations in some cohorts, which were less marked in fully adjusted models (appendix pp 5, 6, 12–15).

Systolic blood pressure was positively and linearly related to maternal age (figure 1; appendix pp 5, 16), but this was non-significant after adjustment for socioeconomic factors. Fasting glucose showed a U-shaped association with maternal age (figure 1; appendix p 5), which remained after full adjustment. Mean fasting glucose was higher by 0·05 mmol/L (95% CI −0·01 to 0·10) in offspring of mothers aged 19 years and younger and 0·06 mmol/L (−0·01 to 0·12) in offspring of mothers aged 35 years and older. This association did not change much after further adjustment for birthweight. Substantial site–sex heterogeneity was noted, due to more linear associations in some cohorts (appendix p 17). None of the body composition or other cardiometabolic outcomes was related to maternal age (table 3; appendix pp 5, 6).

**Figure 2:**
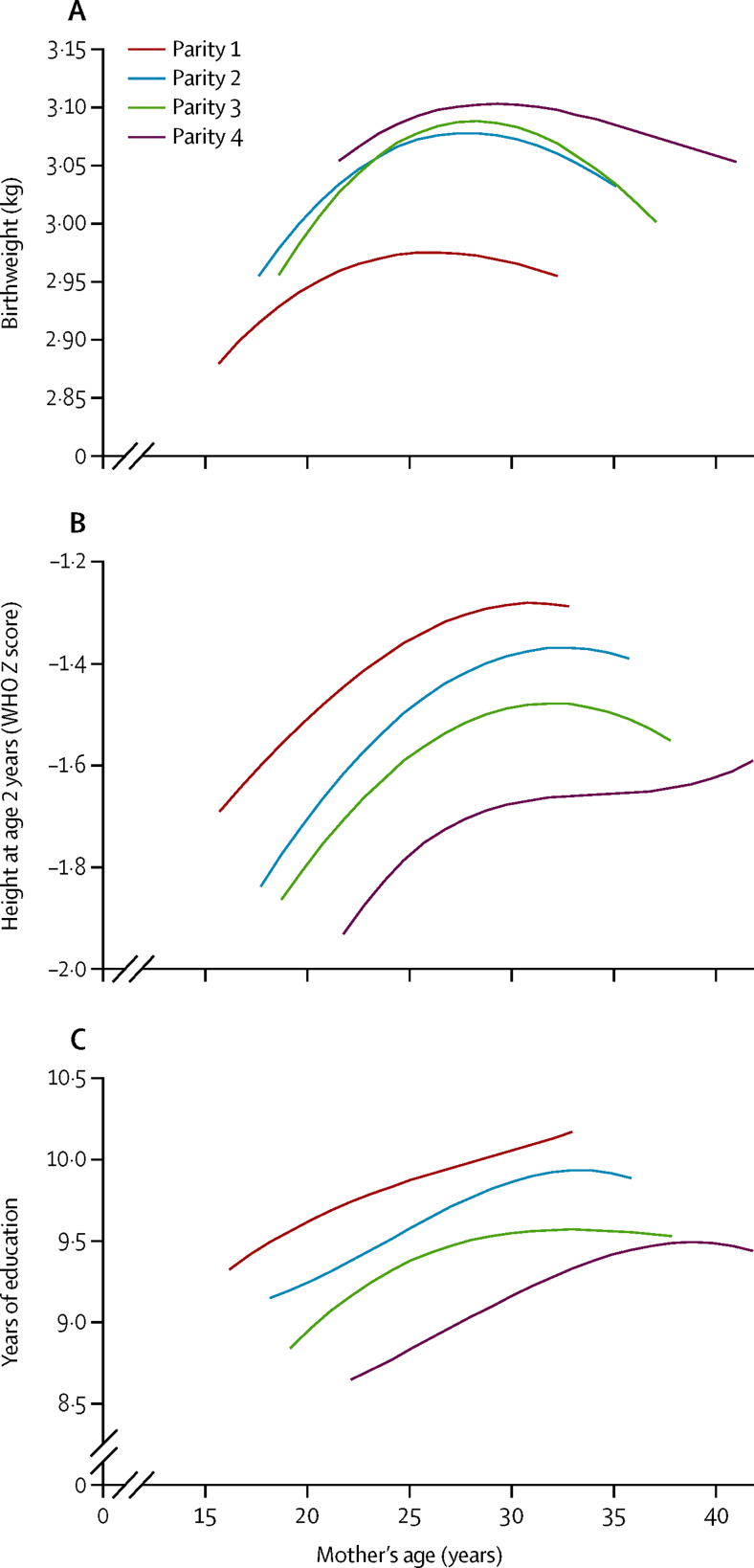
Relation between maternal age and parity and birthweight, 2-year height *Z* score, and years of schooling (adjusted for maternal socioeconomic status and height) Outcomes (birthweight, height *Z* score at age 2 years, and schooling) were adjusted for study site and sex. Within each parity group (1, 2, 3, and 4+), cubic regression was used to model how maternal age was associated with each outcome. The fitted regression lines were plotted for the central 95% of maternal ages for the parity group.

The change in associations between maternal age and outcomes after adjustment for parity suggested strong effects of parity itself. Figure 2 shows these effects on birthweight, 2-year HAZ, and schooling. At any parity, birthweight increased with maternal age up to a threshold around 25–30 years and then decreased, whereas 2-year HAZ and schooling increased up to around 30–35 years, and remained high. At any maternal age, higher parity was associated with higher birthweight (especially for the difference between parity 1 and 2), but lower 2-year HAZ and years of schooling. Higher parity was not associated with lower gestational age or higher risk of preterm birth in young or older mothers.

## Discussion

With data from five population-based cohorts in LMICs, this study, to our knowledge, is one of the largest relating maternal age to birth and childhood outcomes with adjustment for multiple confounders, and the first to relate maternal age to adult outcomes (panel). Children of young mothers and mothers of advanced age had an increased risk of low birthweight and preterm birth, stunting in infancy, short adult height, poor schooling, and higher adult fasting glucose concentrations. The disadvantages of young maternal age were independent of socioeconomic status, height, breastfeeding duration, and parity. With the exception of preterm birth and adult plasma glucose concentration, the disadvantages associated with older maternal age were greatly reduced after adjustment for covariables, especially parity, and older maternal age was associated with distinct advantages for children's height and schooling. Apart from glucose, maternal age was unrelated to offspring cardiometabolic risk factors or body composition.

Children of mothers aged 19 years and younger had a 20–30% increased risk of low birthweight and preterm birth. This finding is consistent with a large body of evidence showing that teenage motherhood is detrimental to the newborn.^1–12,36^ Studies that subcategorised teenage births showed the most adverse effects at the youngest ages.^2–4,6,9,11^ The mechanisms for these effects are important for policy;^8^—for example, if they are explained by socioeconomic factors, interventions that simply delay age at marriage might have little effect. In our study, adjustment for socioeconomic factors attenuated the associations, but they remained significant. This suggests that there are biological or behavioural or a combination of both factors that are not captured by maternal height, breastfeeding duration, or parity. This finding is supported by those noted in different settings.^1–12^ Several recent studies in the UK and the USA have reported that the increased risk of preterm birth among teenage mothers is greater for second-time than for first-time mothers.^37,38^ The reasons for this are unknown, but in a high-income setting, having more than one birth as a teenager could identify a group with high-risk behaviours. We did not note evidence of a similar association in our data from LMICs.

Children of mothers younger than 19 years had a 30–40% increased risk of 2-year stunting and of failure to complete secondary schooling. These associations remained after full adjustment, although residual confounding cannot be ruled out. The associations with poorer child nutritional status, especially stunting, are consistent with other studies.^39^ The effects could be mediated by maternal inexperience, absence of autonomy leading to suboptimum feeding, and hygiene and health-care seeking behaviours,^3^ which we did not measure. Children of young mothers in LMICs have increased mortality, sometimes increasing with the age of the child, suggesting effects of the post natal environment.^3,9^ Studies from HICs have also shown poorer school attainment, cognitive function, or both in children of teenage mothers, independent of socioeconomic factors,^20,40–42^ possibly because of poorer care and stimulation. No previous publications have assessed schooling in relation to maternal age from LMICs.

In recognition of the disadvantages of young motherhood, most countries have a minimum legal age for marriage, but it is often ignored. In many LMICs, 20–30% of girls aged 15–19 years are married, and in some 40–50%.^36,40^ Early marriage has implications for women themselves, truncating their education and violating their human rights.^36,43^ The situation is improving in many places, but worsening in others, and the disparity in teenage fertility rates between HICs and LMICs is widening.^25^ Our findings emphasise the importance of continued and strengthened measures to discourage early marriage and childbirth.

Independent of confounders, and consistent with other data from both HICs and LMICs,^16–18^ mothers 35 years or older had a 30% increased risk of preterm birth. However, in our fully adjusted data, older maternal age was an advantage for the child's nutritional status, schooling, and adult height. These associations were strong (the fully adjusted OR for non-completion of secondary education was 0·59) and have not been reported before from LMICs. They could be related to improved child-rearing practices by more experienced or empowered women.^40–42^

We noted a U-shaped association between maternal age and fasting glucose concentration in the adult offspring. Offspring of younger and older mothers had concentrations of roughly 0·05 mmol/L higher than the reference group, an association that was not evident for impaired glucose tolerance or diabetes. This previously unreported association requires replication. The effect in older mothers could be due to obesity, diabetes, or both in pregnancy, but we have no data to examine this. Offspring body composition and other cardiometabolic risk markers were unrelated to maternal age.

Strengths of the study are a large sample size, information about confounding factors recorded prospectively, and comparable outcomes in all sites. We did not know maternal age at menarche and so could not examine effects of gynaecological age. We did not have data for maternal smoking, diet, and supplement use, other aspects of infant or childhood diet (apart from breastfeeding), and paternal factors, and so residual confounding cannot be ruled out. We cannot exclude confounding by unmeasured genetic or familial factors, which have been recently shown in a large Swedish cohort using sibling comparisons and cousin comparisons to account for an association between young maternal age and attention-deficit hyperactivity disorder in the children.^44^ Adult outcomes were measured with different techniques in the different cohorts, although all were accepted methods; these differences could contribute additional noise in the data, which would tend to reduce associations. Other limitations were losses to follow-up and missing data. 2-year outcomes were available for nearly 80% of the cohort in Brazil and the Philippines, but only 45–50% in Guatemala (where many children were enrolled after age 2 years), India, and South Africa. Percentages for adult blood pressure were greater than 70% in Brazil, 50% in Guatemala, the Philippines, and South Africa, and 14% in India. Comparisons between participants and non-participants showed differences among cohorts in maternal, child, and adult measurements that varied in strength and direction; we adjusted for each maternal factor independently within each cohort to allow for the varied ways in which they were related to outcomes.

Children of teenage mothers in LMICs are disadvantaged at birth and during childhood and have reduced human capital. Measures to prevent young motherhood should be strengthened and young mothers should be helped to improve their children's nutrition and education. Our data show that children of older mothers are at increased risk of preterm birth, and might have increased plasma glucose concentrations in adult life. However, at a given socioeconomic level and parity, children of older mothers have advantages in terms of childhood nutrition and educational attainment.

## Figures and Tables

**Table 1:** Maternal age, birth, child, and adult outcomes, socioeconomic variables and maternal height, breastfeeding, and parity in the five cohorts

			**Brazil**		**Guatemala**		**India**		**Philippines**		**South Africa**	
			Mean (SD) or n (%)	N	Mean (SD) or n (%)	N	Mean (SD) or n (%)	N	Mean (SD) or n (%)	N	Mean (SD) or n (%)	N
Original cohort			..	5913	..	2392	..	7530	..	3080	..	3273
Maternal age			..	5912	..	2344	..	5395	..	3080	..	3271
Maternal age and any outcome of interest			..	5910	..	1942	..	5203	..	3078	..	3270
Maternal age (years)			25·8 (6·1)	5910	27·2 (7·2)	1942	26·0 (5·2)	5203	26·3 (6·0)	3078	26·0 (6·1)	3270
Child outcomes												
	Birthweight (g)		3190 (568)	5804	3048 (492)	973	2792 (440)	4832	2988 (438)	3029	3070 (513)	3265
	Low birthweight		640 (11·0%)	5804	136 (14·0%)	973	1393 (28·8%)	4832	448 (14·8%)	3029	420 (12·9%)	3265
	Small for gestational age		683 (14·9%)	4596	233 (31·1%)	748	361 (40·9%)	883	735 (24·5%)	2999	453 (14·3%)	3166
	Gestation (weeks)		39·3 (1·9)	4600	39·3 (3·0)	851	38·9 (2·6)	937	38·7 (2·2)	3048	38·1 (1·9)	3169
	Preterm birth		289 (6·3%)	4600	109 (12·8%)	851	141 (15·0%)	937	466 (15·3%)	3048	388 (12·2%)	3169
	Height (*Z*) at 2 years		−0·67 (1·25)	4835	−2·91 (1·15)	1079	−2·01 (1·23)	3697	−2·41 (1·15)	2504	−1·16 (1·12)	1804
	Weight-for-height (*Z*) at 2 years		0·56 (0·99)	4832	−0·28 (0·90)	1076	−0·66 (1·03)	3666	−0·62 (0·96)	2502	0·19 (1·35)	1803
	Stunted at 2 years		667 (13·8%)	4835	858 (79·5%)	1079	1841 (49·8%)	3697	1558 (62·2%)	2504	372 (20·6%)	1804
	Wasted at 2 years		40 (0·8%)	4832	52 (4·8%)	1076	362 (9·9%)	3666	182 (7·3%)	2502	86 (4·8%)	1803
	Height (*Z*) MC		−0·66 (1·11)	4651	−2·42 (0·97)	998	−1·98 (1·06)	3197	−2·05 (0·94)	2261	−0·91 (0·93)	1804
	Weight-for-height (*Z*) MC		0·63 (1·01)	4648	0·32 (0·83)	998	−0·17 (0·88)	3173	−0·81 (0·89)	2261	0·18 (1·03)	1804
	Stunted mid-childhood		505 (10·9%)	4651	672 (67·3%)	998	1547 (48·4%)	3197	1215 (53·7%)	2261	212 (11·8%)	1804
	Wasted mid-childhood		15 (0·3%)	4648	2 (0·2%)	998	72 (2·3%)	3173	195 (8·6%)	2261	29 (1·6%)	1804
Adult outcomes												
	Age (years)		22·7 (0·4)	4193	32·4 (4·1)	1297	29·1 (1·3)	1038	21·2 (0·9)	2040	18·1 (0·5)	1989
	Attained schooling (years)		9·3 (3·2)	4076	4·7 (3·4)	1458	13·2 (3·4)	1038	10·0 (3·0)	2069	11·1 (1·5)	2036
	Failed to finish secondary school		1974 (48·4%)	4076	1369 (93·9%)	1458	145 (14·0%)	1038	613 (29·6%)	2069	781 (38·4%)	2036
	Height (cm)											
		Men	173·7 (6·9)	2207	162·8 (6·0)	552	169·3 (6·4)	590	163·0 (5·9)	1079	171·0 (7·5)	933
		Women	160·8 (6·2)	1979	150·6 (5·6)	608	154·3 (5·1)	446	151·2 (5·5)	958	159·7 (6·3)	1000
	Short stature		167 (4·0%)	4186	535 (46·1%)	1160	161 (15·5%)	1036	864 (42·4%)	2037	151 (7·8%)	1933
	Body-mass index (kg/m^2^)		23·6 (4·4)	4185	25·9 (4·4)	1160	24·4 (4·6)	1036	20·6 (3·1)	2032	21·7 (4·2)	1932
	Waist circumference (cm)											
		Men	80·9 (10·1)	2205	86·7 (9·1)	540	88·9 (11·7)	590	72·0 (7·5)	1077	..	..
		Women	74·9 (10·6)	1978	92·4 (12·1)	604	78·6 (12·4)	448	67·6 (7·2)	951	..	..
	Overweight		1190 (28·4%)	4185	603 (52·0%)	1160	465 (44·9%)	1036	176 (8·7%)	2032	330 (17·1%)	1932
	Obese		341 (8·1%)	4185	202 (17·4%)	1160	94 (9·1%)	1036	32 (1·6%)	2032	104 (5·4%)	1932
	Fat mass (kg)											
		Men	12·2 (5·1)	1952	14·0 (6·6)	540	17·2 (6·9)	588	9·7 (4·5)	988	8·7 (5·0)	879
		Women	..	..	22·3 (9·1)	602	20·3 (8·1)	446	15·4 (4·6)	822	19·4 (7·7)	936
	Lean mass (kg)											
		Men	60·3 (9·7)	1952	51·4 (5·4)	540	53·4 (7·8)	588	46·4 (5·8)	988	51·0 (6·7)	879
		Women	..	..	38·9 (3·6)	602	37·6 (5·6)	446	31·0 (4·2)	822	39·0 (5·4)	936
	Body fat (%)											
		Men	16·3 (3·8)	2196	20·5 (6·6)	540	23·5 (6·0)	588	16·7 (5·1)	988	14·1 (5·3)	883
		Women	..	..	35·1 (7·3)	602	33·7 (7·5)	446	32·7 (4·8)	822	32·3 (6·6)	939
	Systolic blood pressure (mm Hg)		117·7 (15·0)	4188	112·1 (12·9)	1248	113·0 (12·2)	1033	105·9 (12·2)	2036	117·6 (10·7)	1871
	Diastolic blood pressure (mm Hg)		73·8 (11·4)	4188	70·8 (9·5)	1248	75·3 (10·0)	1033	72·1 (10·0)	2042	71·4 (8·5)	1871
	High blood pressure		1384 (33·0%)	4188	224 (17·9%)	1248	342 (33·1%)	1033	589 (28·8%)	2044	400 (21·4%)	1871
	Fasting glucose (mmol/L)		5·02 (0·73)	3612	5·20 (1·11)	985	5·43 (1·01)	1013	4·63 (0·55)	1703	4·62 (0·45)	1189
	IFG or diabetes		281 (7·8%)	3618	60 (6·1%)	991	184 (18·1%)	1014	16 (0·9%)	1703	10 (0·8%)	1194
Covariates												
	Mother married		5422 (91·8%)	5905	1557 (91·5%)	1701	5200 (100·0%)	5201	2963 (97·5%)	3038	1414 (43·5%)	3248
	Maternal education (years)		6·5 (4·2)	5910	1·3 (1·6)	1913	5·1 (4·6)	5203	7·1 (3·3)	3078	9·5 (3·0)	3048
	Maternal wealth		3·3 (1·2)	4988	−0·12 (0·93)	1784	4·1 (1·2)	5190	3·5 (2·6)	3077	3·9 (1·6)	2857
	Ethnic origin											
		White	3457 (75·5%)	4580	..	1942	..	5203	..	3078	207 (6·3%)	3270
		Black	711 (15·5%)	4580	..	1942	..	5203	..	3078	2567 (78·5%)	3270
		Asian	76 (1·7%)	4580	..	1942	5203 (100%)	5203	3078 (100%)	3078	115 (3·5%)	3270
		Other	336 (7·3%)	4580	1942 (100%)	1942	..	5203	..	3078	381 (11·7%)	3270
	Urban score		Urban	..	Rural	..	Urban	..	30·6 (12·6)	3078	Urban	..
	Maternal height (cm)		156·4 (6·0)	5804	148·5 (5·2)	1604	151·8 (5·5)	1296	150·6 (5·0)	3078	158·8 (6·1)	1757
	Breastfeeding											
		None	420 (9·4%)	4489	7 (0·7%)	988	..	..	204 (7·6%)	2668	203 (8·5%)	2390
		<6 months	2562 (57·1%)	4489	40 (4·0%)	988	..	..	397 (14·9%)	2668	745 (31·2%)	2390
		<1 year	690 (15·4%)	4489	149 (15·1%)	988	..	..	353 (13·2%)	2668	368 (15·4%)	2390
		≥1 year	817 (18·2%)	4489	792 (80·2%)	988	..	..	1714 (64·2%)	2668	1074 (44·9%)	2390
	Maternal parity											
		1	2321 (39·3%)	5908	310 (16·0%)	1938	77·2 (17·2%)	4492	686 (22·3%)	3078	1197 (36·6%)	3270
		2	1659 (28·1%)	5908	295 (15·2%)	1938	1140 (25·4%)	4492	693 (22·5%)	3078	988 (30·2%)	3270
		3	965 (16·3%)	5908	272 (14·0%)	1938	1000 (22·3%)	4492	598 (19·4%)	3078	578 (17·7%)	3270
		4+	963 (16·3%)	5908	1061 (54·7%)	1938	1580 (35·2%)	4492	1101 (35·8%)	3078	507 (15·5%)	3270

Breastfeeding duration was not available for India. Imputed values were used for maternal schooling (Brazil, n=7 [0·1%]; Guatemala, n=64 [3·3%]; India, n=9 [0·17%]; Philippines, n=0; South Africa, n=341 [10·4%]) and maternal wealth (Brazil, n=922 [15·6%]; Guatemala, n=158 [8·1%]; India, n=13 [2·5%]); Philippines, n=1 [0·03%]; South Africa, n=413 [12·6%]). Maternal height was missing for 106 in Brazil (1·8%), 338 in Guatemala (17·4%), 3907 in India (75·1%), 0 in the Philippines, and 1513 (46·3%) in South Africa. IFG=impaired fasting glucose. MC=mid-childhood.

**Table 2:** Associations between potential confounding and mediating variables and maternal age and offspring birthweight, gestational age at delivery, size at age 2 years, schooling and adult height, systolic blood pressure, and fasting glucose between cohorts

		**Maternal age (years)**	**Birthweight (g)**	**Gestational age (weeks)**	**Height at 2 years (*Z*)**	**Weight-for-height at age 2 years (*Z*)**	**Schooling (years)**	**Adult height (cm)**	**Systolic blood pressure (mm Hg)**	**Fasting glucose (mmol/L)**
**Maternal schooling (correlation)**										
Brazil		0·06‡	0·09‡	−0·03	0·31‡	0·14‡	0·51‡	0·12‡	−0·01	−0·03
Guatemala		−0·05*	0·08*	−0·01	0·06	0·01	0·28‡	0·12‡	0	−0·06*
India		−0·12‡	0·17‡	−0·02	0·45‡	0·28‡	0·38‡	0·12‡	0	0·02
Philippines		−0·05†	0·05*	0·04	0·33‡	0·13‡	0·46‡	0·14‡	0·04	−0·03
South Africa		−0·15‡	0·06†	0·06†	0·16‡	−0·01	0·21‡	0·05*	−0·09‡	−0·03
**Marital status (mean values)**										
Brazil										
	Married	26·1	3203	39·3	−0·65	0·56	9·4	167·6	117·8	5·03
	Single	22·5‡	3046‡	39·3	−0·91‡	0·53	8·4‡	167·1	116·5	4·99
Guatemala										
	Married	27·9	3075	39·4	−2·88	−0·26	4·7	156·6	112·2	5·23
	Single	26·1†	2896†	38·4*	−3·20*	−0·53†	4·0*	155·8	111·7	5·18
Philippines										
	Married	26·5	2993	38·7	−2·41	−0·62	10·0	157·5	105·8	4·63
	Single	21·8‡	2852†	38·5	−2·34	−0·58	9·9	157·6	105·3	4·45*
South Africa										
	Married	28·4	3098	38·4	−1·02	0·02	11·1	166·1‡	118·1	4·64
	Single	24·1‡	3051†	38·0‡	−1·25‡	0·30‡	11·0	164·6	117·3	4·61
**Wealth (correlation)**										
Brazil		0·22‡	0·11‡	−0·04*	0·34‡	0·17‡	0·47‡	0·10‡	0	−0·06†
Guatemala		0·02	0·11†	−0·01	0·14‡	0·09†	0·23‡	0·10†	0·03	−0·01
India		0·11‡	0·13‡	−0·01	0·23‡	0·17‡	0·22‡	0·05	0·03	0·02
Philippines		0·09‡	0·08‡	0·02	0·26‡	0·15‡	0·24‡	0·12‡	0·04	−0·01
South Africa		0·02	0·02	0·06†	0·19‡	−0·05	0·16‡	0·03	−0·06*	0·03
**Urban (correlation)**										
Philippines		−0·06†	0·04*	−0·01	0·15‡	−0·04	0·20‡	0·04	0·01	−0·02
**Ethnic origin (mean values)**										
Brazil										
	White	26·0	3247‡	39·4	−0·55‡	0·61‡	9·8‡	167·9*	117·3†	5·02
	Black	25·8	3139‡	39·2	−1·10‡	0·44‡	8·2‡	166·7*	119·4†	5·00
	Asian	25·2	3225‡	39·7	−0·98‡	0·38‡	8·3‡	166·1*	115·8†	5·04
	Other	26·3	3207‡	39·3	−0·99‡	0·47‡	7·8‡	167·1*	119·1†	5·13
South Africa										
	White	27·8‡	3197‡	39·0‡	−0·37‡	0·36‡	11·6‡	169·8†	114·3‡	NA
	Black	25·9‡	3077‡	37·9‡	−1·24‡	0·35‡	11·1‡	165·0†	118·0‡	4·62
	Asian	26·3‡	2911‡	38·8‡	−0·77‡	−0·58‡	11·6‡	167·1†	111·0‡	4·86
	Other	25·6‡	3007‡	38·9‡	−1·13‡	−0·51‡	10·7‡	164·9†	116·1‡	4·63
**Maternal height (correlation)**										
Brazil		0	0·18‡	0·04†	0·38‡	0·13‡	0·19‡	0·38‡	0·03	−0·01
Guatemala		−0·01	0·14‡	−0·02	0·30‡	0·07*	0·10‡	0·32‡	0·03	0·01
India		−0·02	0·09‡	0	0·20‡	0·08‡	0·04	0·14‡	−0·03	0·07*
Philippines		0·06†	0·20‡	0·01	0·32‡	0·14‡	0·13‡	0·34‡	0·04	0
South Africa		0·01	0·09‡	0·02	0·16‡	0·03	0·04	0·27‡	0·09‡	0·02*
**Breastfeeding duration (correlation)**										
Brazil		0·09‡	0·09‡	0·04*	−0·01	−0·05‡	0·02	0·02	0·02	0
Guatemala		0·13‡	0·06	0·03	0·13‡	−0·11†	−0·02	0·05	0·07	0·06
Philippines		0·04*	0·05†	0·04*	−0·09‡	−0·12‡	−0·20‡	−0·05*	−0·01	0·03
South Africa		−0·01	0·04	0·01	−0·12‡	0·04	0	0	0·04	−0·04
**Parity (correlation)**										
Brazil		0·53‡	0·07‡	−0·04*	−0·16‡	−0·09‡	−0·22‡	−0·06‡	−0·01	0
Guatemala		0·67‡	0·21‡	0·01	−0·04	0·03	−0·10‡	−0·01	0	0·02
India		0·50‡	0·08‡	0·07*	−0·21‡	−0·11‡	−0·19‡	−0·12‡	−0·03	−0·05
Philippines		0·64‡	0·17‡	0·03	−0·20‡	−0·06†	−0·14‡	−0·03	−0·01	0·04
South Africa		0·68‡	0·08‡	0·06†	−0·06*	−0·02	−0·09‡	0·04	0·10‡	0

All participants in India were married. The Brazilian, Indian, and South African studies were set in exclusively urban areas. The Guatemala study was set in an exclusively rural area. Participants in India and the Philippines were classified as Asian and in Guatemala as other. Breastfeeding duration was not available for India. NA=data not available.

* p<0·05.

† p<0·01.

‡ p<0·001. p values for marital status are from a 2-sample *t* test; those for ethnic origin are from a 3 degrees of freedom *F* test.

**Table 3:** Association between maternal age and low birthweight, preterm birth, 2-year stunting and wasting, non-completion of secondary education, adult overweight or obesity, high blood pressure, and abnormal glucose tolerance

	**Low birthweight**	**Preterm birth**	**2-year stunting**	**2-year wasting**	**Non-completion of secondary schooling**	**Adult overweight or obesity**	**Adult high blood pressure**	**Adult impaired fasting glucose or diabetes**
**Model 1—Maternal age (years; adjusted for sex only)**								
≤19	1·43 (1·26–1·62)	1·44 (1·21–1·72)	1·37 (1·20–1·56)	1·12 (0·86–1·46)	1·32 (1·15–1·51)	1·07 (0·92–1·25)	1·00 (0·87–1·16)	0·87 (0·64–1·18)
20–24	1·00 (Ref)	1·00 (Ref)	1·00 (Ref)	1·00 (Ref)	1·00 (Ref)	1·00 (Ref)	1·00 (Ref)	1·00 (Ref)
25–29	0·83 (0·75–0·92)	0·91 (0·78–1·06)	0·84 (0·76–0·93)	0·91 (0·75–1·11)	0·80 (0·72–0·90)	0·96 (0·84–1·08)	1·05 (0·94–1·19)	0·98 (0·78–1·23)
30–34	0·82 (0·72–0·93)	0·91 (0·77–1·08)	0·93 (0·83–1·04)	1·05 (0·84–1·31)	0·86 (0·76–0·98)	1·06 (0·92–1·21)	1·11 (0·97–1·27)	0·69 (0·52–0·91)
≥35	0·94 (0·81–1·08)	1·30 (1·08–1·57)	1·18 (1·03–1·35)	0·97 (0·74–1·28)	0·99 (0·85–1·16)	1·01 (0·86–1·19)	1·08 (0·92–1·26)	1·06 (0·79–1·41)
p*	<0·0001	0·29	0·02	0·64	<0·0001	0·76	0·17	0·86
p†	<0·0001	<0·0001	<0·0001	0·16	<0·0001	0·74	0·35	0·29
**Model 2 (adjusted for sex and socioeconomic factors)**								
≤19	1·36 (1·19–1·56)	1·35 (1·12–1·62)	1·23 (1·06–1·41)	1·05 (0·80–1·38)	1·13 (0·97–1·31)	1·11 (0·95–1·29)	1·00 (0·86–1·16)	0·87 (0·64–1·19)
20–24	1·00 (Ref)	1·00 (Ref)	1·00 (Ref)	1·00 (Ref)	1·00 (Ref)	1·00 (Ref)	1·00 (Ref)	1·00 (Ref)
25–29	0·87 (0·78–0·97)	0·91 (0·77–1·06)	0·88 (0·79–0·98)	0·95 (0·77–1·16)	0·87 (0·77–0·99)	0·95 (0·84–1·08)	1·04 (0·93–1·18)	0·98 (0·78–1·23)
30–34	0·83 (0·73–0·94)	0·92 (0·77–1·10)	0·87 (0·77–0·99)	0·98 (0·78–1·23)	0·87 (0·76–1·01)	1·05 (0·91–1·21)	1·10 (0·96–1·25)	0·69 (0·52–0·92)
≥35	0·92 (0·79–1·07)	1·28 (1·04–1·56)	0·97 (0·83–1·13)	0·79 (0·59–1·06)	0·88 (0·74–1·05)	1·02 (0·87–1·21)	1·06 (0·90–1·24)	1·05 (0·78–1·43)
p*	<0·0001	0·69	0·0009	0·19	0·001	0·59	0·30	0·88
p†	<0·0001	<0·0001	0·003	0·92	0·10	0·56	0·35	0·22
**Model 5 (adjusted for sex, socioeconomic factors, maternal height, breastfeeding duration, and parity)**								
≤19	1·18 (1·02–1·36)	1·26 (1·03–1·53)	1·46 (1·25–1·70)	1·11 (0·83–1·49)	1·38 (1·18–1·62)	1·10 (0·94–1·29)	0·95 (0·81–1·11)	0·87 (0·63–1·20)
20–24	1·00 (Ref)	1·00 (Ref)	1·009 (Ref)	1·00 (Ref)	1·00 (Ref)	1·00 (Ref)	1·00 (Ref)	1·00 (Ref)
25–29	0·96 (0·85–1·08)	0·96 (0·81–1·14)	0·72 (0·63–0·81)	0·84 (0·68–1·04)	0·71 (0·62–0·81)	0·98 (0·86–1·12)	1·07 (0·94–1·21)	0·98 (0·77–1·25)
30–34	0·94 (0·82–1·09)	0·96 (0·79–1·18)	0·63 (0·54–0·73)	0·82 (0·63–1·06)	0·63 (0·53–0·74)	1·12 (0·96–1·30)	1·13 (0·97–1·31)	0·69 (0·51–0·94)
≥35	1·03 (0·86–1·23)	1·33 (1·05–1·67)	0·64 (0·54–0·77)	0·66 (0·48–0·90)	0·59 (0·48–0·71)	1·13 (0·94–1·36)	1·08 (0·90–1·29)	1·03 (0·74–1·44)
p*	0·14	0·37	<0·0001	0·01	<0·0001	0·44	0·18	0·94
p†	0·001	0·0005	<0·0001	0·32	0·0008	0·69	0·09	0·16

Data are odds ratio (95% CI).

* p=p value for linear association.

† p=p value for quadratic association. p values derived with logistic regression, with maternal age and maternal age^2^ as continuous variables and outcomes as binary.

## Panel Research in context

**Systematic review**

We searched PubMed for studies describing the relation between maternal age and birth outcomes, child nutritional status, attained schooling, and adult body composition and cardiometabolic risk factors in the offspring, published between Jan 1, 1990, and Jan 1, 2014, in English language. There is a large literature documenting an increased risk of adverse birth outcomes when the mother is very young (<19 years) or of advanced age (>35 years). Young motherhood is also associated with increased undernutrition and poor educational attainment in the children. This literature, especially that relating to advanced maternal age, is overwhelmingly dominated by studies from high-income countries. Few studies in any setting have examined advanced maternal age as a predictor of childhood outcomes, and none have examined young or advanced maternal age as predictors of adult body composition and cardiometabolic risk factors.

**Interpretation**

Our study pooled data from five birth cohorts in low-income and middle-income countries (LMICs) creating a large sample size (roughly 17 000 for birth outcomes, about 13 000 for child outcomes, and about 10 000 for adult outcomes). All studies collected data for maternal age at childbirth (exposure), and longitudinal measurements of the children's weight and height, attained schooling, and adult body composition and cardiometabolic risk factors (outcomes). They also collected data for maternal socioeconomic status, parity, height, and duration of breastfeeding, which might confound the relation of maternal age with these outcomes. We identified that young maternal age was associated with an increased risk of low birthweight, preterm and smallness-for-gestational-age births, stunting and wasting in infancy and childhood, and failure to complete secondary education. The associations were attenuated by adjustment for confounding factors, but remained significant, which suggests that they result from biological or behavioural immaturity of the mother. The results reinforce the arguments for LMICs to strengthen measures to prevent early marriage and childbearing. We confirmed that advanced maternal age was associated with an increased risk of preterm birth, independent of confounders. After adjustment for confounders, older maternal age was associated with improved child nutritional status and further schooling, suggesting that, other factors being equal, children of more mature mothers are advantaged nutritionally and educationally. Adult body composition and blood pressure were unrelated to maternal age, but fasting plasma glucose concentrations were increased in offspring of young and old mothers. This finding has not been reported before and should be replicated in other studies, but suggests that there could be effects of maternal age on fetal and child development, which programme adult glucose metabolism.

## References

[1] Paranjothy S, Broughton H, Adappa R, Fone D (2009). Teenage pregnancy: who suffers?. Arch Dis Child.

[2] DuPlessis HM, Bell R, Richards T (1997). Adolescent pregnancy; understanding the impact of age and race on outcomes. J Adolesc Health.

[3] LeGrand TK, Mbacke CS (1993). Teenage pregnancy and child health in the urban Sahel. Stud Fam Plann.

[4] Conde-Agudelo A, Belizan JM, Lammers C (2005). Maternal-perinatal morbidity and mortality associated with adolescent pregnancy in Latin America: cross-sectional survey. Am J Obstet Gynecol.

[5] Markovitz BP, Cook R, Flick LH, Leet TL (2005). Socioeconomic factors and adolescent pregnancy outcomes; distinctions between neonatal and postneonatal deaths?. BMC Public Health.

[6] Sharma V, Katz J, Mullany LC (2008). Young maternal age and the risk of neonatal mortality in rural Nepal. Arch Paediatr Adolesc Med.

[7] DeVienne CM, Creveuil C, Dreyfus M (2009). Does young maternal age increase the risk of adverse obstetric, fetal and neonatal outcomes: a cohort study. Eur J Obstet Gynecol Reprod Biol.

[8] Lawlor DA, Mortensen L, Andersen AM (2011). Mechanisms underlying the associations of maternal age with adverse perinatal outcomes: a sibling study of 264 695 Danish women and their firstborn offspring. Int J Epidemiol.

[9] Restrepo-Mendez MC, Barros AJD, Santos IS (2011). Childbearing during adolescence and offspring mortality: findings from three population-based cohorts in southern Brazil. BMC Public Health.

[10] Alam N (2000). Teenage motherhood and infant mortality in Bangladesh: maternal age-dependent effect of parity one. J Biosoc Sci.

[11] Gibbs C, Wendt A, Peters S, Hogue CJ (2012). The impact of early age at first childbirth on maternal and infant health. Paediatr Perinat Epidemiol.

[12] Borja J, Adair LS (2003). Assessing the net effect of young maternal age on birth weight. Am J Hum Biol.

[13] Wambach KA, Cole C (2000). Breastfeeding and adolescents. J Obstet Gynecol Neonatal Nurs.

[14] Scholl TO, Hediger ML (1993). A review of the epidemiology of nutrition and adolescent pregnancy: maternal growth during pregnancy and its effects on the fetus. J Am Coll Nutr.

[15] Carolan M, Frankowska D (2011). Advanced maternal age and adverse perinatal outcome; a review of the evidence. Midwifery.

[16] Ngowa JDK, Ngassam A, Dohbit JS, Nzedjom C, Kasia JM (2013). Pregnancy outcome at advanced maternal age in a group of African women in two teaching hospitals in Yaounde, Cameroon. Pan Afr Med J.

[17] Kenny LC, Lavender T, McNamee R, O'Neill SM, Mills T, Khashan AS (2013). Advanced maternal age and adverse pregnancy outcome: evidence from a large contemporary cohort. PLoS One.

[18] Newburn-Cook CV, Onyskiw JE (2005). Is older maternal age a risk factor for pre-term birth and fetal growth restriction; a systematic review. Health Care Women Int.

[19] Victora CG, Adair L, Fall C, the Maternal and Child Undernutrition Study Group (2008). Maternal and child undernutrition: consequences for adult health and human capital. Lancet.

[20] Shaw M, Lawlor D, Najman JM (2006). Teenage children of teenage mothers: psychological, behavioural and health outcomes from an Australian prospective longitudinal study. Soc Sci Med.

[21] Whincup PH, Cook DG, Shaper AG (1989). Early influences on blood pressure: a study of children aged 5-7 years. BMJ.

[22] Lawlor DA, Naiman JM, Sterne J, Williamns GM, Ebrahim S, Davey Smith G (2004). Associations of parental, birth and early life characteristics with systolic blood pressure at 5 years of age: findings from the Mater-University study of pregnancy and its outcomes. Circulation.

[23] Gillman MW, Rich-Edwards JW, Rifas-Shiman SL (2004). Maternal age and other predictors of newborn blood pressure. J Pediatr.

[24] Roberts RJ, Leary SD, Smith GD, Ness AR and ALSPAC study team (2005). Maternal age in pregnancy and offspring blood pressure in childhood in the Avon Longitudinal Study of Parents and Children (ALSPAC). J Hum Hypertens.

[25] United Nations; Dept of Economic and Social Affairs, Population Division (2009). World Fertility Patterns. http://www.un.org/esa/population/publications/worldfertility2009/worldfertility2009.htm.

[26] Richter LM, Victora CG, Hallal PC (2012). Cohort profile: the Consortium of Health-Orientated Research in Transitioning Societies. Int J Epidemiol.

[27] Ballard JL, Novak KK, Driver M (1979). A simplified score for assessment of fetal maturation of newly born infants. J Pediatr.

[28] Williams RL, Creasy RK, Cunningham GC, Hawes WE, Norris FD, Tashiro M (1982). Fetal growth and perinatal viability in California. Obstet Gynecol.

[29] Kuzawa C, Hallal P, Adair L (2012). Birth weight, postnatal weight gain and adult body composition in five low and middle income countries. Am J Hum Biol.

[30] Adair LS, Martorell R, Stein AD (2009). Size at birth, weight gain in infancy and childhood, and adult blood pressure in five low and middle income country cohorts: When does weight gain matter?. Am J Clin Nutr.

[31] International Diabetes Federation (2006). The IDF consensus worldwide definition of the metabolic syndrome.

[32] Norris SA, Osmond C, Gigante D (2012). Size at birth, weight gain in infancy and childhood, and adult diabetes risk in five low- or middle-income country birth cohorts. Diabetes Care.

[33] WHO (1999). Definition, diagnosis and classification of diabetes mellitus and its complications. Report of a WHO consultation. Part 1: Diagnosis and Classification of Diabetes Mellitus.

[34] Adair L, Dahly D (2007). Quantifying the urban environment; a scale measure of urbanicity outperforms the rural-urban dichotomy. Soc Sci Med.

[35] Armitage P, Berry G, Matthews JNS (2002). Statistical methods in medical research.

[36] United Nations Population Fund (UNFPA) (2012). Marrying too young; end child marriage.

[37] Smith GCS, Pell JP (2001). Teenage pregnancy and risk of adverse perinatal outcomes associated with first and second births: population based retrospective cohort study. BMJ.

[38] Khashan AS, Baker PN, Kenny LC (2010). Preterm birth and reduced birthweight in first and second teenage pregnancies; a register-based cohort study. BMC Pregnancy Childbirth.

[39] Raj A, Saggurti N, Winter M (2010). The effect of maternal child marriage on morbidity and mortality of children under 5 in India: cross-sectional study of a nationally representative sample. BMJ.

[40] Boden JM, Fergusson DM, John Horwood L (2008). Early motherhood and subsequent life outcomes. J Child Psychol Psychiatry.

[41] Fergusson DM, Woodward LJ (1999). Maternal age and educational and psychological outcomes in early adulthood. J Child Psychol Psychiatry.

[42] Morinis J, Carson C, Quigley MA (2013). Effect of teenage motherhood on cognitive outcomes in children: a population-based cohort study. Arch Dis Child.

[43] United Nations (2000). World Marriage Patterns. http://www.un.org/esa/population/publications/worldmarriage/worldmarriagepatterns2000.pdf.

[44] Chang Z, Lichtenstein P, D'Onofrio BM (2014). Maternal age at childbirth and risk for ADHD in offspring: a population-based cohort study. Int J Epidemiol.

