# Supplementary material for: Association between maternal age at childbirth and child and adult outcomes in the offspring: a prospective study in five low-income and middle-income countries (COHORTS collaboration)
Source: Lancet Glob Health. 2015 Jul;3(7):e341–422. doi: 10.1016/S2214-109X(15)00038-8 (PMC4547329; doi:10.1016/S2214-109X(15)00038-8)

THE LANCET Global Health

Supplementary appendix

This appendix formed part of the original submission and has been peer reviewed. We post it as supplied by the authors.

Supplement to: Fall CHD, Sachdev HS, Osmond C, et al, and the COHORTS investigators. Association between maternal age at childbirth and child and adult outcomes in the offspring: a prospective study in five low-income and middle-income countries (COHORTS collaboration). *Lancet Glob Health* 2015; published online May 19. http://dx.doi.org/10.1016/S2214-109X(15)00038-8.

EXTRA SUPPLEMENTARY MATERIAL Table 1: Details of the five COHORTS studies at recruitment

Study	Design	Cohort inception	Initial sample	Comments
Pelotas, Brazil	Prospective cohort	1982	5913	Enrolled all children born in the city's maternity hospitals (>99% of all births) during 1982. All social classes included.
INTCS Guatemala	Community trial	1969-77	2392	Intervention trial of a high-energy and protein supplement. All children <7 y in 1969 and all born 1969-77 were enrolled and followed until age 7 or until the study ended in 1977. Data were collected on mothers during pregnancy and breast feeding periods.
New Delhi, India	Prospective cohort	1969-72	7530	Pregnancies were identified in a population of married women living in a defined area of Delhi, and the newborns were enrolled and followed. Primarily middle-class sample.
CLHNS, Philippines	Prospective cohort	1983-84	3080	Pregnant women living in 33 randomly selected neighbourhoods; 75% urban. First data collection at 30 weeks gestation. All social classes included.
Bt20, South Africa	Prospective cohort	1990	3273	Pregnant women with a gestational age of 26-32 weeks living in a delimited urban geographical area. Predominantly poor, black sample.

EXTRA SUPPLEMENTARY MATERIAL Table 2: Derivation of the wealth index in each of the five cohorts

Brazil: Data were obtained when the child was aged 2 years. Five variables were used in a factor analysis to obtain the wealth index: 1) House construction type (brick/wood/brick and wood/apartment building/slum house/other); 2) Number of bedrooms in the house; 3) Access to water (tap inside the household/tap outside the household/no tap); 4) Access to toilet facilities (flush toilet/pit latrine/none); 5) Presence in the household of the following assets/items, each scored 0/1: radio, television, refrigerator, gas stove, wood burning stove.

Guatemala: Data were obtained during a survey at recruitment of each family into the trial. The following variables were used in a principal components analysis and the first component was retained as the wealth index: 1) Live in a formal house (0/1); 2) House construction type (high-quality wall, roofing, flooring, 0/1 for each); 3) Separate kitchen in the house (0/1); 4) Number of rooms in the house divided by the total number of occupants; 5) Formal cooking medium (0/1); 6) Electricity in the house (0/1); 7) Own the house (0/1); 8) Own the plot of land for the house (0/1); 9) Access to a latrine/toilet (0/1); 10) Access to drinking water from a well (0/1); 11) Ownership of the following assets/items, each scored 0/1: radio, record-player, bicycle, sewing machine, refrigerator, television, motorcycle, automobile. The method has been described in full in: Maluccio JA, Murphy A, Yount KM. Research note: a socio-economic index for the INCAP longitudinal study 1969-1977. Food and Nutrition Bulletin 2005; 26 Supplement 1: S120-124.

India: The information was obtained during pregnancy. We used two variables relating to housing: 1) Ownership (Government/Owned/Rented/Free/Other) and 2) Type of house (thatched hut/ masonry build/apartment building/bungalow (separate house)). Variable 1 was re-coded into owned or not owned, and combined with type of housing to create a score from 1-8 as follows: 1) not owned thatched hut; 2) owned thatched hut; 3) not owned masonry build; 4) owned masonry build; 5) not owned apartment; 6) owned apartment; 7) not owned bungalow; 8) owned bungalow.

Philippines: Data were collected during pregnancy. We used ownership of the home and land, and a number of household assets owned, each scored 0/1, including electrical appliances (air-conditioner, electric fan, electric iron, television, other), boat, vehicle (car, jeepney, truck, other), bicycle, animals (water buffalo, cow, goat, pig), and furniture (bed, dining set, living room suite). These were subjected to principal components analysis, and the first component retained as the wealth index.

South Africa: Data were collected between birth and 2 years. The following information was used to create the wealth index, using a simple sum (0-8): 1) Type of home (1=house/flat; 0=other); 2) Ownership of the home (1=own; 0=don’t own); 3) Electricity in the home (0/1); 4) Ownership of the following items, each scored 0/1: television, car, refrigerator, washing machine, telephone.

EXTRA SUPPLEMENTARY MATERIAL Table 3: Maternal age distribution; number (%) in maternal age categories in each cohort

a) Everyone in the dataset with a known maternal age N=20,002

Maternal age	Brazil	Guatemala	India	Philippines	South Africa	All
**<15**	**13** (<1)	**10** (<1)	**0** (0)	**1** (<1)	**5** (<1)	**29** (<1)
**15-19**	**899** (15)	**356** (15)	**439** (8)	**382** (12)	**479** (15)	**2555** (13)
**20-24**	**1842** (31)	**656** (28)	**1889** (35)	**977** (32)	**1010** (31)	**6374** (32)
**25-29**	**1599** (27)	**493** (21)	**1770** (33)	**862** (28)	**860** (26)	**5584** (28)
**30-34**	**973** (17)	**393** (17)	**877** (16)	**532** (17)	**583** (18)	**3358** (17)
**35-39**	**442** (8)	**296** (13)	**314** (6)	**240** (8)	**273** (8)	**1565** (8)
**≥40**	**144** (2)	**140** (6)	**106** (2)	**86** (3)	**61** (2)	**537** (3)
**All**	**5912** (100)	**2344** (100)	**5395** (100)	**3080** (100)	**3271** (100)	**20002** (100)

b) If offspring height at 2 years known, N=13,919

**<15**	**10** (<1)	**3** (<1)	**0** (0)	**1** (<1)	**3** (<1)	**17** (<1)
**15-19**	**708** (15)	**151** (14)	**247** (7)	**301** (12)	**299** (17)	**1706** (12)
**20-24**	**1467** (30)	**288** (27)	**1238** (34)	**787** (31)	**546** (30)	**4326** (31)
**25-29**	**1298** (27)	**244** (23)	**1228** (33)	**712** (28)	**453** (25)	**3935** (28)
**30-34**	**842** (17)	**196** (18)	**661** (18)	**434** (17)	**317** (18)	**2450** (18)
**35-39**	**386** (8)	**132** (12)	**245** (7)	**198** (8)	**151** (8)	**1112** (8)
**≥40**	**124** (3)	**65** (6)	**78** (2)	**71** (3)	**35** (2)	**373** (3)
**All**	**4835** (100)	**1079** (100)	**3697** (100)	**2504** (100)	**1804** (100)	**13919** (100)

c) If adult systolic blood pressure known, N=10,376

**<15**	**9** (<1)	**6** (<1)	**0** (0)	**1** (<1)	**1** (<1)	**17** (<1)
**15-19**	**596** (14)	**179** (14)	**73** (7)	**259** (13)	**312** (17)	**1419** (14)
**20-24**	**1294** (31)	**336** (27)	**339** (33)	**636** (31)	**556** (30)	**3161** (31)
**25-29**	**1134** (27)	**272** (22)	**292** (28)	**569** (28)	**481** (26)	**2748** (27)
**30-34**	**729** (17)	**219** (18)	**206** (20)	**345** (17)	**335** (18)	**1834** (18)
**35-39**	**315** (8)	**168** (14)	**92** (9)	**167** (8)	**153** (8)	**895** (9)
**≥40**	**111** (3)	**68** (5)	**31** (3)	**59** (3)	**33** (2)	**302** (3)
**All**	**4188** (100)	**1248** (100)	**1033** (100)	**2036** (100)	**1871** (100)	**10376** (100)

There were small numbers in the 2 extreme maternal groups; therefore the two youngest and two oldest groups were merged for subsequent presentation in tables and figures. The 20-24y age group was the most numerous, and was used as the reference group in regression analyses.

EXTRA SUPPLEMENTARY MATERIAL Table 4: Comparison between cohort members who did and did not participate at 2-year and adult follow-ups

Measurement	2 year follow-up					Adult follow-up				
	Mean for those followed up – mean for those not followed up					Mean for those followed up – mean for those not followed up				
	Brazil	Guatemala	India	Philippines	S. Africa	Brazil	Guatemala	India	Philippines	S. Africa
**Number followed up / not followed up**	4835/1077	1079/1265	3697/1698	2504/576	1804/1467	4186/1726	1160/1184	1036/4359	2037/1043	1933/1338
**Mother**										
**Education (years)**	0·71***	-0·05	0·77***	-0·49**	0·61***	0·03	-0·04	-0·33*	-0·32*	0·34**
**Married (%)**	4·0***	0·9	0·0	0·2	-10·4***	2·0**	0·5	0·0	0·5	-14·7***
**Wealth index (z)**	0·59***	-0·04	0·46***	0·37**	0·61***	0·04	-0·11*	0·42***	0·41***	0·17**
**Height (cm)**	0·57**	0·10	0·38	0·15	-0·69*	0·22	-0·31	-0·63**	0·07	-0·53
**Parity**	-0·01	0·20***	0·16***	0·13*	-0·11**	-0·02	0·19***	0·20***	0·03	-0·06
**Child: birth**										
**Sex (% male)**	-4·6**	-1·3	-0·6	1·9	1·2	-4·6**	7·6***	-6·1***	0·1	0·9
**Birth weight (g)**	213***	-29	99***	66**	29	129***	69*	31	56**	-19
**Gestational age (weeks)**	0·52***	0·47	-0·18	0·14	0·07	0·33***	0·22	1·16	0·05	-0·17*
**Child: age 2 years**										
**HAZ**	—	—	—	—	—	0·00	-0·22**	-0·10*	0·11*	-0·14*
**WHZ**	—	—	—	—	—	0·04	-0·10	-0·01	0·10*	-0·03
**Child: adult**										
**Height (cm)**	0·20	-0·28	-0·29	-1·34*	-0·53	—	—	—	—	—
**Education (years)**	0·63***	0·18	-0·75	-0·58	0·16*	—	—	—	—	—
**Systolic blood pressure (mm Hg)**	-0·92	-0·43	0·74	1·08	-0·36	—	—	—	—	—

* P<0·05; ** p<0·01. *** p<0·001

EXTRA SUPPLEMENTARY MATERIAL Table 5: Associations of maternal age with child and continuous variable adult outcomes

	**MODEL 1 Adjusted for age, sex and site only**				**MODEL 2 Model 1 + adjusted for socio-economic variables**				**MODEL 3 Model 2 + adjusted for maternal height**				**MODEL 4 Model 3 + adjusted for breastfeeding duration**				**MODEL 5 Model 4 + adjusted for maternal parity**			
	p-lin	p-quad	h-lin	h-quad	p-lin	p-quad	h-lin	h-quad	p-lin	p-quad	h-lin	h-quad	p-lin	p-quad	h-lin	h-quad	p-lin	p-quad	h-lin	h-quad
**Birth outcomes**																				
Birth weight	+***	-***	0·09	0·8	+***	-***	0·2	0·8	+0·00	-***	0·2	0·9	+***	-***	0·2	0·9	+0·00	-***	***	0·3
Gestation	-0·4	-***	0·001	0·2	-0·06	-***	0·001	0·4	-0·06	-***	0·001	0·3	-0·06	-***	0·002	0·4	-***	-***	0·008	0·5
**Child outcomes**																				
Height at 2y	+***	-***	***	***	+0·00	-***	0·1	0·01	+***	-***	0·01	0·004	+0·00	-***	0·001	0·005	+***	-***	0·4	0·01
Weight for height at 2y	+0·2	-***	0·8	0·01	+0·1	-0·03	0·3	0·04	+0·1	-0·04	0·3	0·04	+0·03	-0·04	0·3	0·06	+***	-0·007	0·7	0·06
Height at mc	+***	-***	0·003	***	+***	-0·002	0·2	0·001	+***	-0·004	0·04	***	+***	-0·004	0·08	***	+***	-***	0·1	0·001
Weight for height at mc	+0·1	-0·5	0·04	0·2	+0·3	+0·9	0·3	0·1	+0·3	+0·9	0·4	0·1	+0·3	+0·9	0·2	0·09	+0·3	+0·8	0·02	0·03
**Adult outcomes**																				
Schooling	+0·00	-***	***	***	+0·01	-0·5	0·002	0·4	+0·00	-0·6	0·003	0·3	+0·00	-0·6	0·006	0·3	+***	-0·002	***	0·3
Height	+***	-***	0·009	***	+0·00	-0·02	0·4	0·004	+***	-0·04	0·2	0·002	+***	-0·04	0·3	0·002	+***	-0·002	0·2	0·01
Body mass index	-0·2	+0·09	0·1	1·0	-0·1	+0·05	0·5	1·0	-0·1	+0·04	0·5	1·0	-0·1	+0·04	0·6	1·0	+0·7	+0·2	0·2	0·8
Percent fat	-0·4	-0·7	**	0·3	-0·5	+0·4	0·1	0·3	-0·6	+0·3	0·1	0·2	-0·6	+0·3	0·1	0·2	+0·09	+0·7	0·4	0·1
Waist circumference	-0·2	+0·1	0·1	0·7	-0·2	+0·07	0·4	0·7	-0·3	+0·03	0·4	0·6	-0·3	+0·03	0·6	0·6	+0·3	+0·09	0·3	0·3
Systolic blood pressure	+0·04	+0·6	0·2	0·4	+0·2	+0·6	0·4	0·2	+0·1	+0·5	0·4	0·1	+0·2	+0·6	0·4	0·1	+0·1	+1·0	0·3	0·03
Diastolic blood pressure	+0·1	+0·9	0·7	0·2	+0·3	+0·9	0·8	0·2	+0·3	+0·8	0·8	0·2	+0·4	+0·9	0·9	0·2	+0·07	-0·9	0·6	0·2
Fasting glucose	+0·9	+0·00	0·09	0·02	+0·8	+0·00	0·2	0·01	+0·8	+0·00	0·2	0·01	-1·0	+0·00	0·3	0·01	+0·8	+0·00	0·08	0·01

Data are presented from multiple linear regression analyses pooled across all 5 sites. p-lin = p-value for a linear trend (sign indicates direction); p-quad = p-value for a quadratic trend (sign indicates direction); h-lin = p-value for heterogeneity in the linear trend across sites; h-quad = p-value for heterogeneity in the quadratic trend across sites. *** = <0·001. mc=mid-childhood

EXTRA SUPPLEMENTARY MATERIAL Table 6: Associations of maternal age with birth, child and adult outcomes that are dichotomous variables.

Outcome	MODEL 1 Adjusted for age, sex and site only				MODEL 2 Model 1 + adjusted for socio-economic variables				MODEL 3 Model 2 + adjusted for maternal height				MODEL 4 Model 3 + adjusted for breastfeeding duration				MODEL 5 Model 4 + adjusted for maternal parity			
	p-lin	p-quad	h-lin	h-quad	p-lin	p-quad	h-lin	h-quad	p-lin	p-quad	h-lin	h-quad	p-lin	p-quad	h-lin	h-quad	p-lin	p-quad	h-lin	h-quad
**Birth outcomes**																				
Low birth weight	-***	+***	0·06	1·0	-***	+***	0·009	0·9	-***	+***	0·01	0·9	-***	+***	0·01	0·9	-0·1	+0·001	0·006	0·8
Preterm birth	-0·3	+***	0·06	0·8	-0·7	+***	0·08	0·8	-0·7	+***	0·09	0·8	-0·8	+***	0·1	0·9	+0·4	+***	0·3	0·8
Small for gestational age	-***	+***	0·004	0·06	-***	+***	0·02	0·04	-***	+0·001	0·01	0·06	-***	+0·001	0·03	0·06	-0·2	+0·2	0·02	0·01
**Child outcomes**																				
Stunted at 2y	-0·02	+***	0·1	***	-0·001	+0·003	0·4	0·1	-0·001	+0·01	0·2	0·09	-0·003	+0·02	0·1	0·09	-***	+***	0·9	0·03
Wasted at 2y	-0·6	+0·2	0·8	0·01	-0·2	+0·9	0·7	***	-0·2	+1·0	0·7	0·001	-0·2	+0·9	0·7	***	-0·01	+0·3	1·0	0·007
Stunted at mc	-0·07	+***	0·2	***	-0·005	+0·3	0·3	***	-0·005	+0·7	0·1	***	-0·002	+0·8	0·2	***	-***	+0·002	0·5	***
Wasted at mc	+0·6	-0·1	1·0	0·03	+0·4	-0·08	1·0	0·03	+0·4	-0·06	1·0	0·03	+0·5	-0·06	1·0	0·03	+0·6	-0·08	0·4	***
**Adult outcomes**																				
Failed to complete 2^0 educ	-***	+***	***	0·09	-0·001	+0·1	0·2	0·3	-0·001	+0·1	0·2	0·3	-0·002	+0·1	0·3	0·3	-***	+***	***	0·2
Short stature	-0·3	+0·5	0·04	0·01	-0·1	-1·0	0·4	0·05	-0·09	-0·4	0·4	0·07	-0·06	-0·5	0·4	0·08	-0·001	-0·8	0·3	0·05
Overweight or obese	-0·8	+0·7	0·1	0·9	-0·6	+0·6	0·5	0·8	-0·6	+0·5	0·5	0·7	-0·5	+0·5	0·6	0·7	+0·4	+0·7	0·4	0·5
Obese	-0.3	+0.7	0.04	0.5	-0.2	+0.6	0.03	0.8	-0.2	+0.6	0.03	0.8	-0.2	+0.7	0.03	0.9	-0.2	+0.7	0.2	0.8
Elevated blood pressure	+0·2	-0·4	0·6	0·3	+0·3	-0·3	0·5	0·3	+0·3	-0·3	0·5	0·3	+0·6	-0·3	0·6	0·3	+0·2	-0·09	0·4	0·4
IFG or diabetes	-0·9	+0·3	0·2	0·1	-0·9	+0·2	0·2	0·1	-0·9	+0·2	0·2	0·2	-0·8	+0·3	0·3	0·1	-0·9	+0·2	0·7	0·1

Data are presented from multiple logistic regression analyses pooled across all 5 sites. p-lin = p-value for a linear trend (sign indicates direction); p-quad = p-value for a quadratic trend (sign indicates direction); h-lin = p-value for heterogeneity in the linear trend across sites; h-quad = p-value for heterogeneity in the quadratic trend across sites. *** = <0·001. mc = mid-childhood. IFG = impaired fasting glucose.

FIGURE LEGENDS

EXTRA SUPPLEMENTARY MATERIAL Figure 1: Associations of maternal age with birth weight, gestational age, 2-year height and weight-for-height Z scores, years of schooling, adult height, systolic blood pressure and fasting plasma glucose. This is equivalent to Figure 1 in the main paper, but controls for sibships in Guatemala and India using linear mixed modelling.

EXTRA SUPPLEMENTARY MATERIAL Figure 2: Associations of maternal age with birth weight by cohort

EXTRA SUPPLEMENTARY MATERIAL Figure 3: Associations of maternal age with gestational age at delivery, by cohort

EXTRA SUPPLEMENTARY MATERIAL Figure 4: Associations of maternal age with 2-year height-for-age Z-score by cohort

EXTRA SUPPLEMENTARY MATERIAL Figure 5: Associations of maternal age with 2-year weight-for-height Z-score by cohort

EXTRA SUPPLEMENTARY MATERIAL Figure 6: Associations of maternal age with attained schooling (years) by cohort

EXTRA SUPPLEMENTARY MATERIAL Figure 7: Associations of maternal age with adult height (cm) by cohort

EXTRA SUPPLEMENTARY MATERIAL Figure 8: Associations of maternal age with adult systolic blood pressure (mmHg) by cohort

EXTRA SUPPLEMENTARY MATERIAL Figure 9: Associations of maternal age with adult plasma fasting glucose concentration (mmol/l) by cohort

Common footnote to ESM Figures 1-9: Each point represents the amount by which the outcome differs from the value obtained for offspring of mothers aged 20-24 years. These estimates are shown with 95% confidence intervals. Three of the five models discussed in the text are included. Model 1is adjusted for sex and age. Model 2 is further adjusted for maternal marital status, schooling, wealth, race and for urbanicity. Models 3 and 4, in which further adjustment is made for maternal height and breastfeeding duration respectively, are not shown because the results were similar to Model 2. Model 5 is further adjusted for parity. Two p-values are shown: "*p lin*" is from a test for linear trend in the outcome with mother's age; "*p quad*" is from a test for quadratic trend in the outcome with mother's age. Both p values are derived using maternal age as a continuous variable.

EXTRA SUPPLEMENTARY MATERIAL Figure 1: Associations of maternal age with birth weight, gestational age, 2-year height and weight-for-height Z scores, years of schooling, adult height, systolic blood pressure and fasting plasma glucose. This is equivalent to Figure 1 in the main paper, but controls for sibships in Guatemala and India using linear mixed modelling.

The largest discrepancy from Figure 1 is for adult height at maternal age <19 years, for which the coefficient of -0.84cm was reduced to -0.70cm when controlling for sibships.

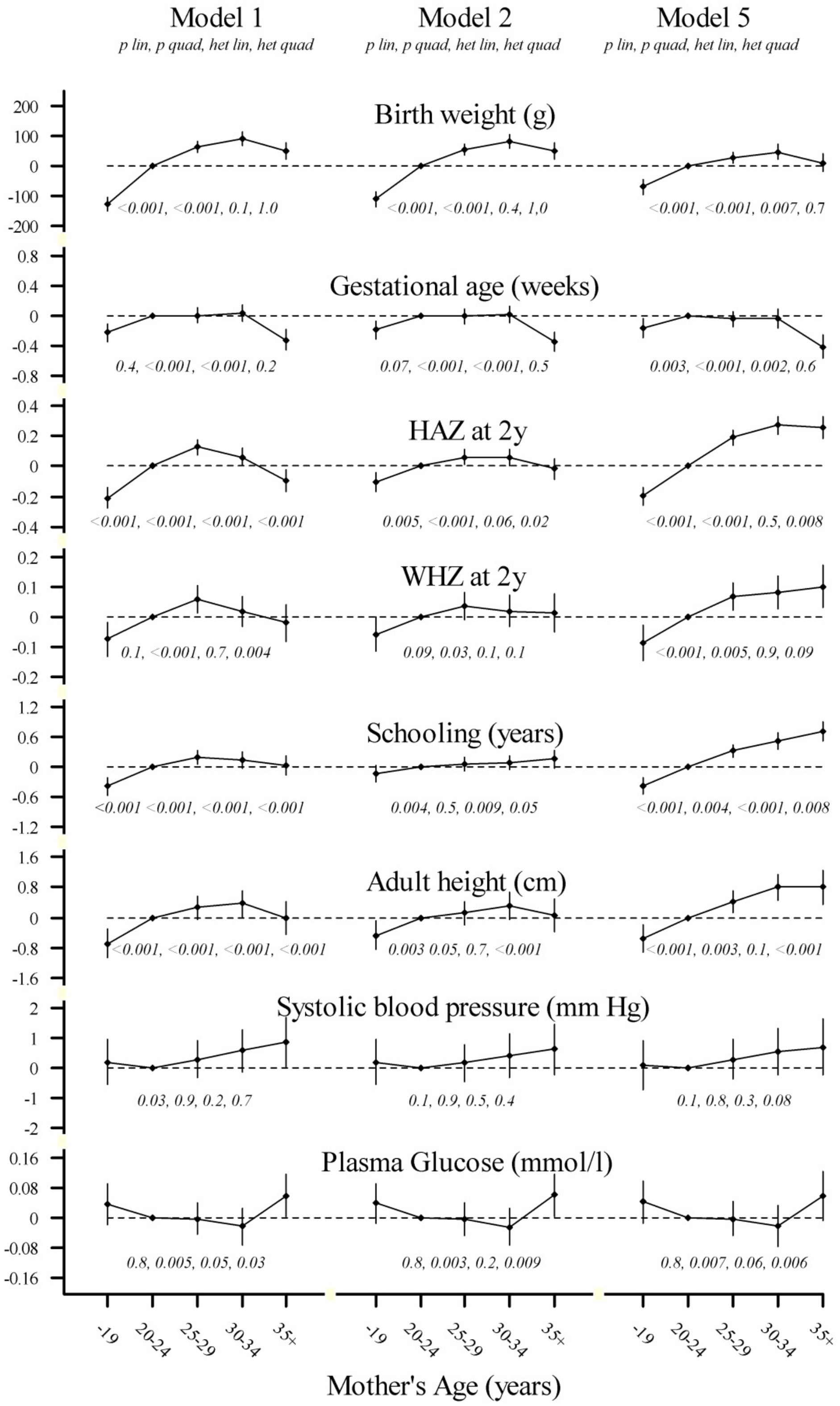

EXTRA SUPPLEMENTARY MATERIAL Figure 2: Associations of maternal age with birth weight by cohort

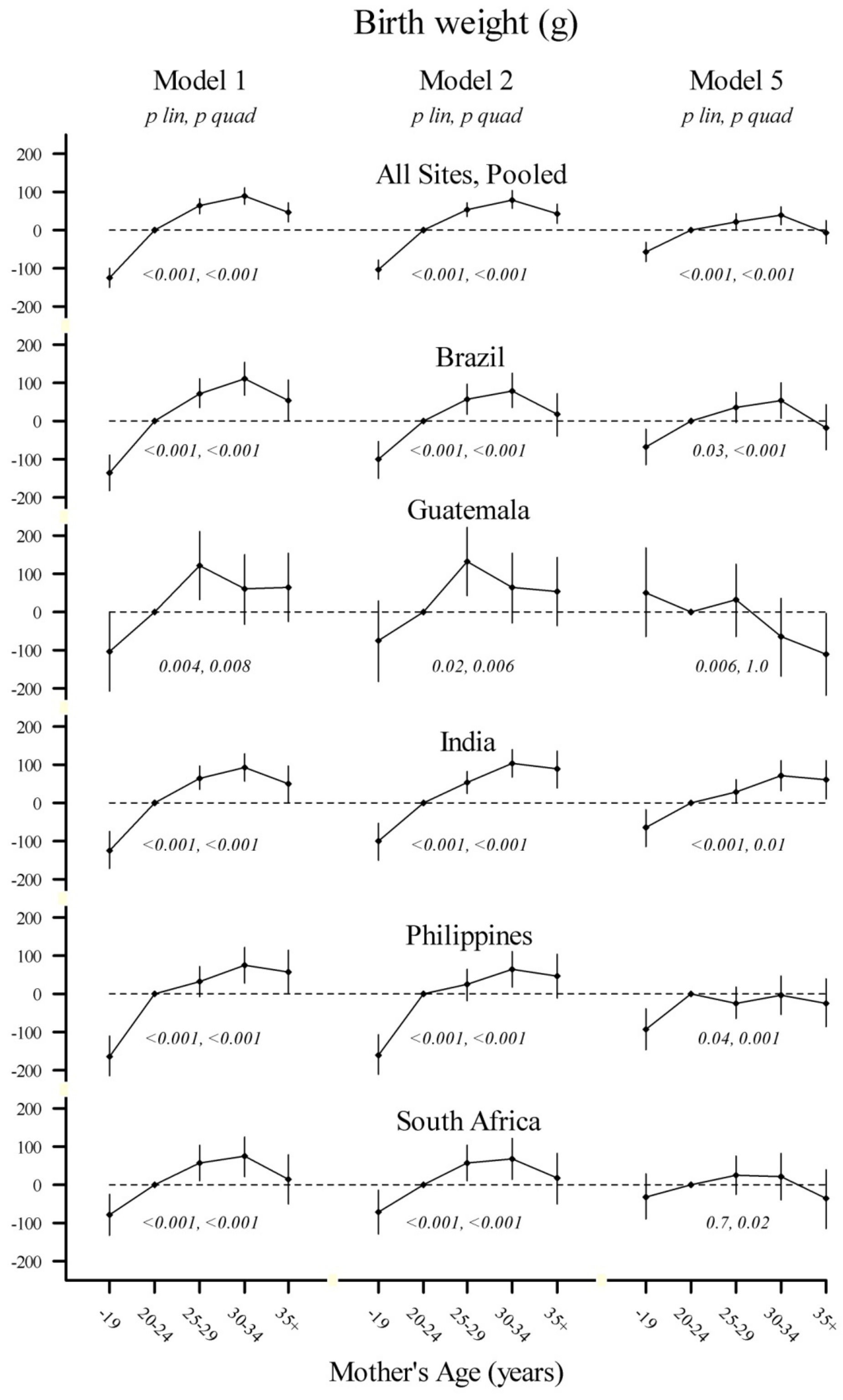

EXTRA SUPPLEMENTARY MATERIAL Figure 3: Associations of maternal age with gestational age at delivery, by cohort

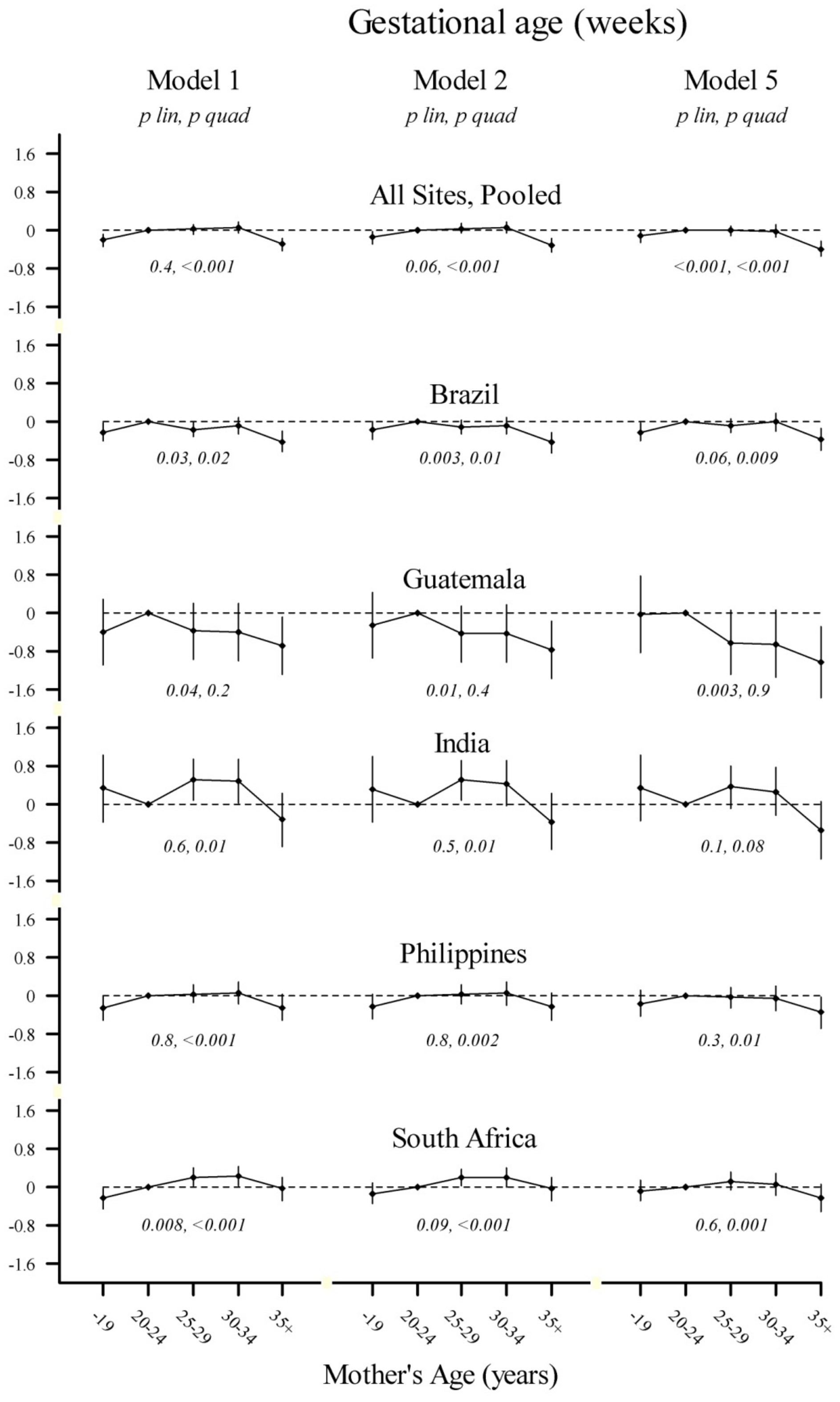

EXTRA SUPPLEMENTARY MATERIAL Figure 4: Associations of maternal age with 2-year height-for-age Z-score by cohort

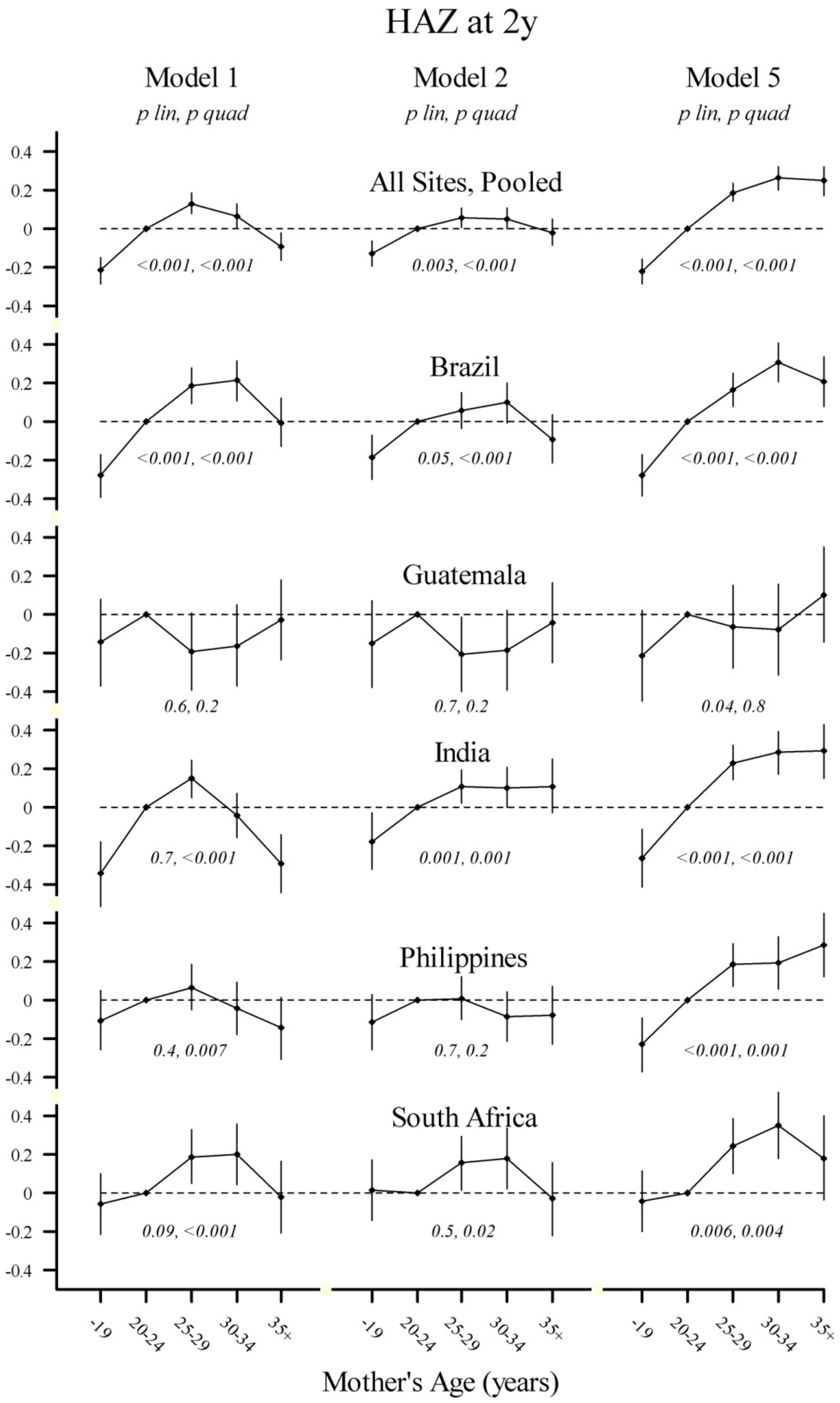

EXTRA SUPPLEMENTARY MATERIAL Figure 5: Associations of maternal age with 2-year weight-for-height Z-score by cohort

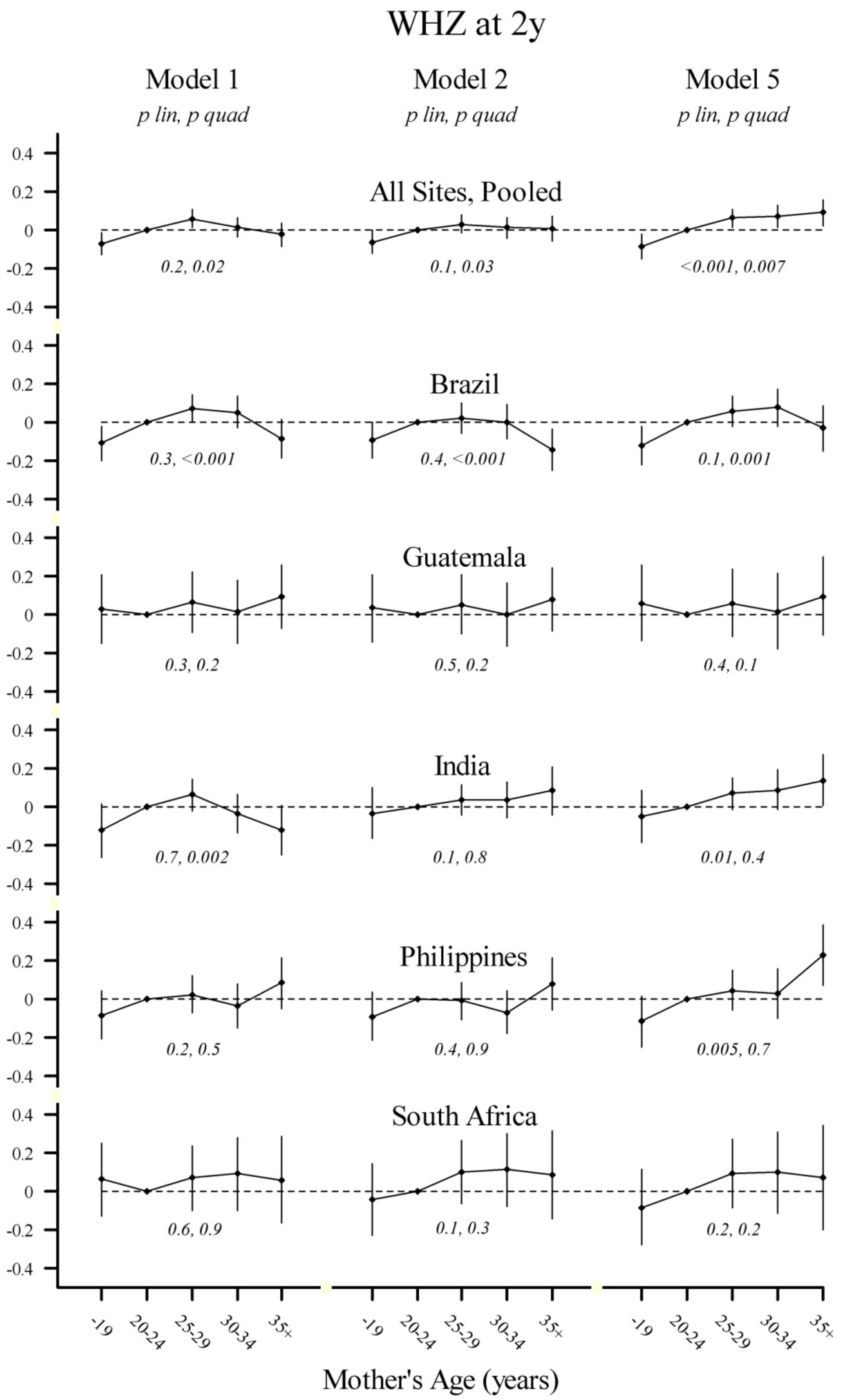

EXTRA SUPPLEMENTARY MATERIAL Figure 6: Associations of maternal age with attained schooling (years) by cohort

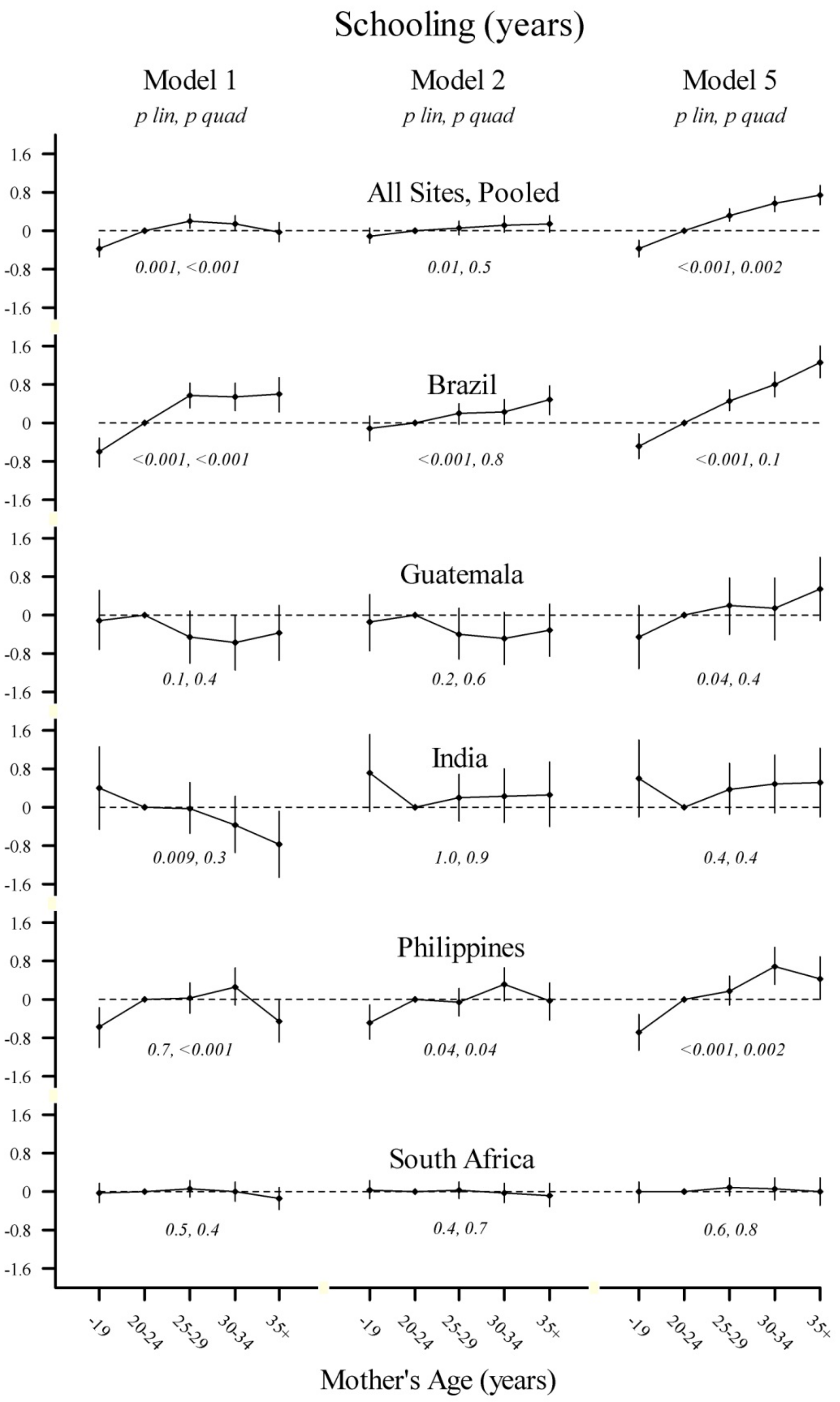

EXTRA SUPPLEMENTARY MATERIAL Figure 7: Associations of maternal age with adult height (cm) by cohort

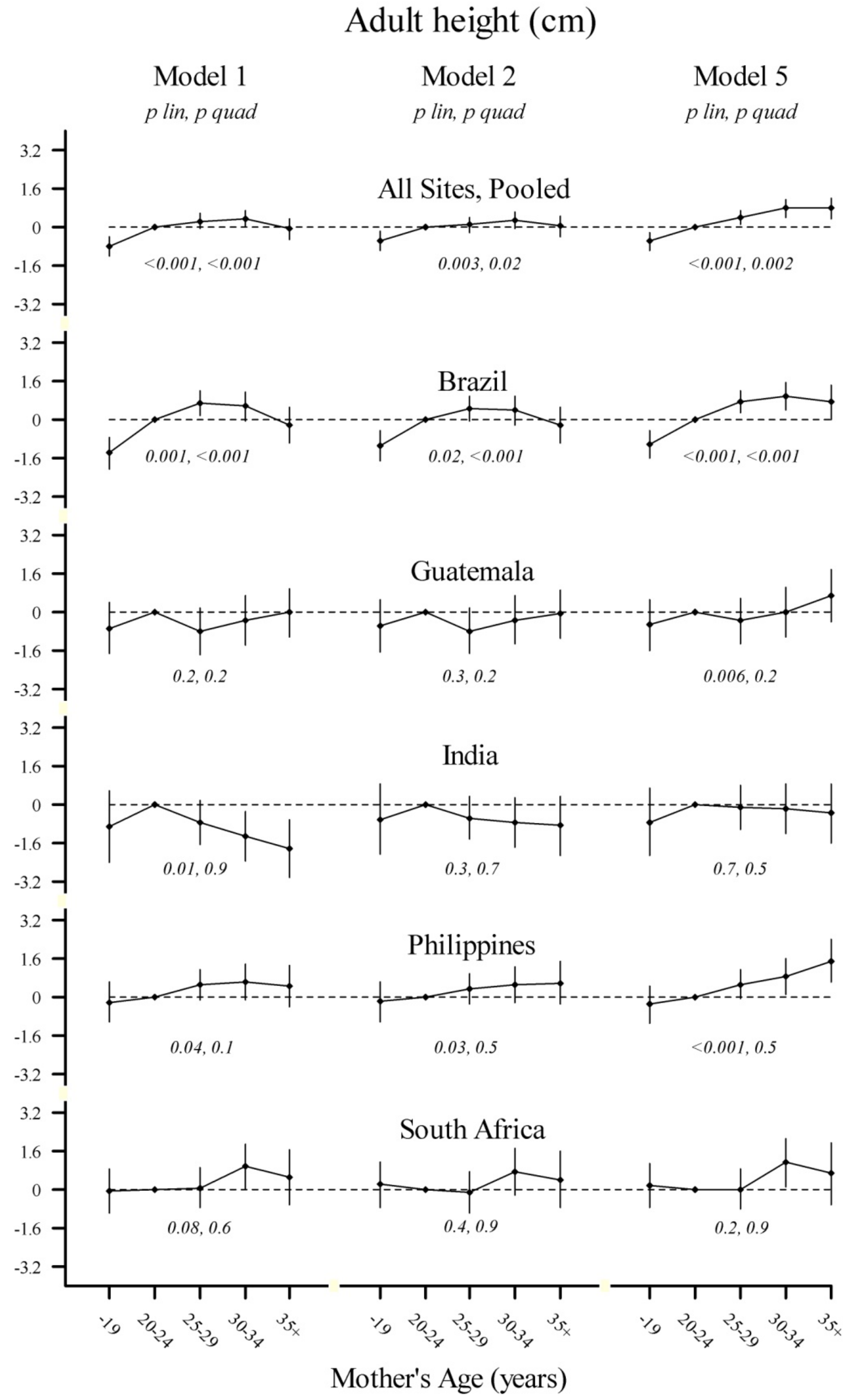

EXTRA SUPPLEMENTARY MATERIAL Figure 8: Associations of maternal age with adult systolic blood pressure (mmHg) by cohort

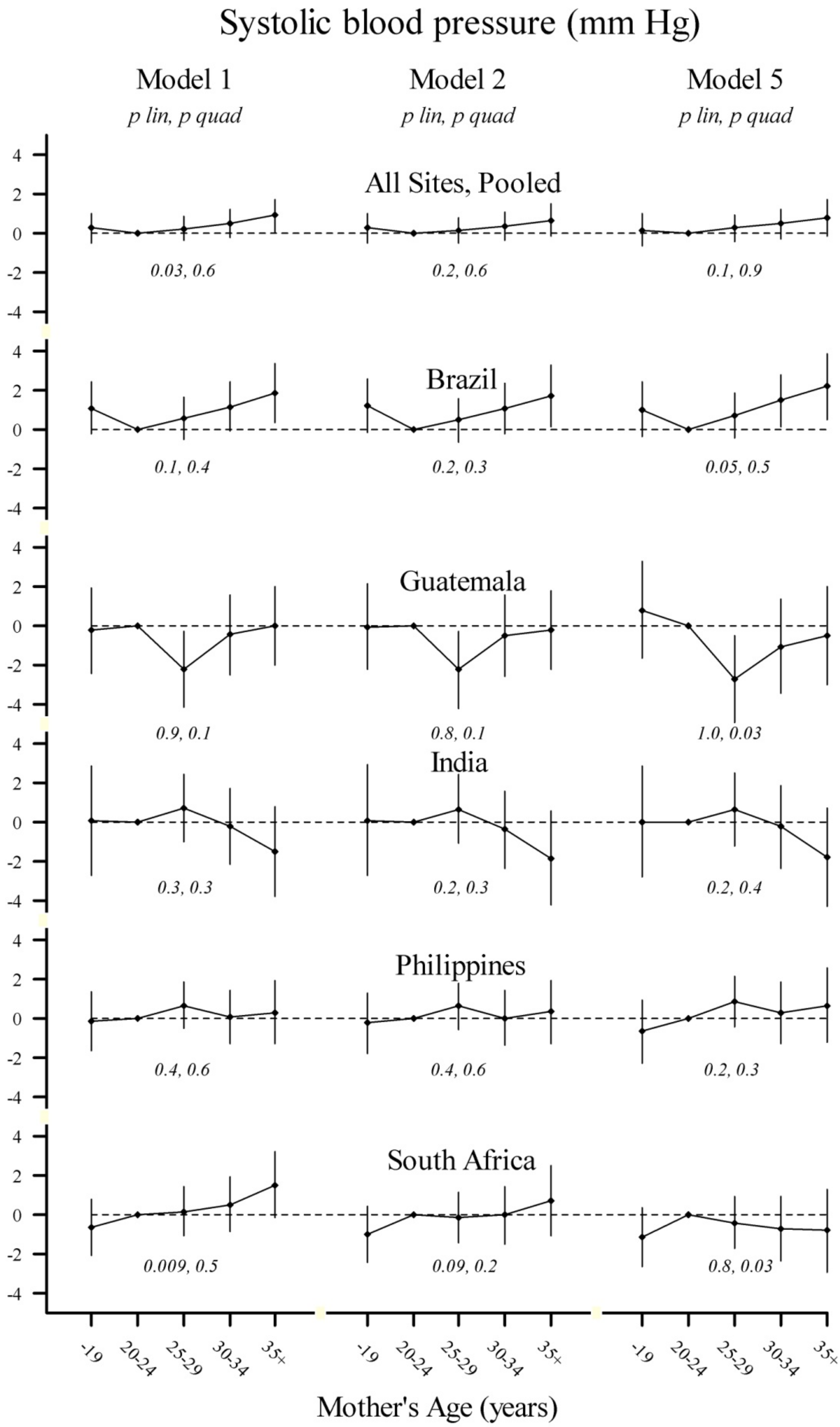

EXTRA SUPPLEMENTARY MATERIAL Figure 9: Associations of maternal age with adult plasma fasting glucose concentration (mmol/l) by cohort

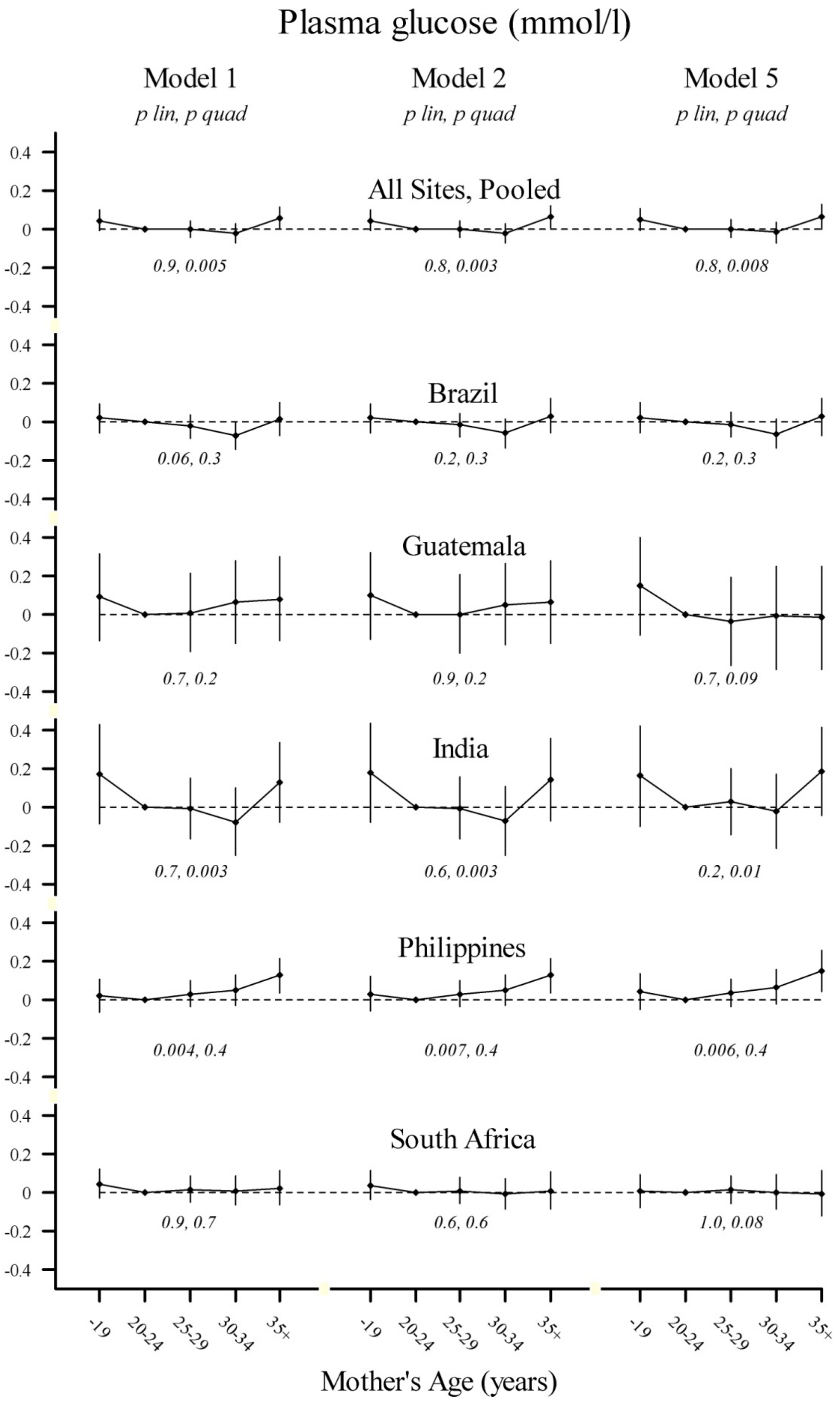

Supplement: Supplementary appendix [file mmc1.pdf]
